# A prognosis model for predicting immunotherapy response of esophageal cancer based on oxidative stress-related signatures

**DOI:** 10.32604/or.2023.030969

**Published:** 2023-11-15

**Authors:** JING GUO, CHANGYONG TONG, JIANGUANG SHI, XINJIAN LI, XUEQIN CHEN

**Affiliations:** 1 Department of Thoracic Surgery, The First Affiliated Hospital of Ningbo University, Ningbo, China; 2 Department of Chinese Traditional Medicine, The First Affiliated Hospital of Ningbo University, Ningbo, China

**Keywords:** Esophageal carcinoma, OS, Prognosis, Risk markers, Immune cell infiltration

## Abstract

Oxidative stress (OS) is intimately associated with tumorigenesis and has been considered a potential therapeutic strategy. However, the OS-associated therapeutic target for esophageal squamous cell carcinoma (ESCC) remains unconfirmed. In our study, gene expression data of ESCC and clinical information from public databases were downloaded. Through LASSO-Cox regression analysis, a risk score (RS) signature map of prognosis was constructed and performed external verification with the GSE53625 cohort. The ESTIMATE, xCell, CIBERSORT, TIMER, and ImmuCellAI algorithms were employed to analyze infiltrating immune cells and generate an immune microenvironment (IM). Afterward, functional enrichment analysis clarified the underlying mechanism of the model. Nomogram was utilized for forecasting the survival rate of individual ESCC cases. As a result, we successfully constructed an OS-related genes (OSRGs) model and found that the survival rate of high-risk groups was lower than that of low-risk groups. The AUC of the ROC verified the strong prediction performance of the signal in these two cohorts further. According to independent prognostic analysis, the RS was identified as an independent risk factor for ESCC. The nomogram and follow-up data revealed that the RS possesses favorable predictive value for the prognosis of ESCC patients. qRT-PCR detection demonstrated increased expression of MPC1, COX6C, CYB5R3, CASP7, and CYCS in esophageal cancer patients. In conclusion, we have constructed an OSRGs model for ESCC to predict patients’ prognosis, offering a novel insight into the potential application of the OSRGs model in ESCC.

## Introduction

Esophageal squamous cell carcinoma (ESCC) is a commonly observed clinical digestive tract tumor, with a reported incidence ranking 8th and a mortality ranking 6th in terms of tumors worldwide [1]. ESCC include neoplasms of esophageal glandular epithelium (EAC) as well as squamous carcinoma of upper esophagus, which exhibit differences in etiology, incidence and clinical features according to research [2]. Most of Chinese patients are diagnosed with ESCC [3]. The overall survival rate of advanced ESCC within years is highly unsatisfactory (18.8%) [4]. Furthermore, ESCC patients often present without significant clinical features in the early stage, and the majority are diagnosed in the late stage, which significantly detracts from their prognosis [5]. As medical advancements continue to be made, an increasing number of treatment options are becoming available for ESCC, including surgical resection, radiotherapy, chemotherapy, and targeted therapy. Immunosuppressants such as CTLA-4, as well as immune system modulators, have also shown considerable clinical efficacy [6]. However, the treatment conditions for most ESCC patients do not meet the requirements, underscoring the urgent need to clarify the molecular mechanisms of cancer immunotherapy and identify effective biomarkers of immune checkpoint inhibitors.

Oxidative stress (OS) is a pathological phenomenon stemming from an imbalance between the synthesis of oxidants and antioxidants. The primary cause is an imbalance of free radicals (produced during oxidation) with the body’s ability to scavenge these free radicals under certain pathological conditions [7]. Various reactive oxygen species (ROS), reportedly produced by the human body, are by-products of aerobic metabolism, sustained pressure, and exposure to ultraviolet rays or X rays [8]. Moreover, ROS plays a critical role in cell signal transduction and the regulation of growth factors, cytokines, hormones, transcription, ion transport, immune regulation, nerve regulation, and apoptosis [9]. The accumulation of high-level ROS is a potential key driver of carcinogenesis and cancer development [10]. Klaunig [11] reported higher ROS levels in leukemia cells than in normal cells, suggesting this as a novel method for targeting tumor cells. However, Liu et al. [12] revealed that the progression of esophageal squamous cell carcinoma can be suppressed by modulating IFI6 depletion to alter the functional dynamics of mitochondria and stimulate the endoplasmic reticulum. Thus, regulating ROS production and the expression of antioxidant oncogenes can influence the apoptosis pathway and ultimately control the progression of ESCC [13]. Redox-targeted pathways and transcription factors hold enormous potential in disease prevention and therapy [14]. Currently, numerous drugs are known to affect the redox cell signaling pathway [15]. However, this does not yet resolve the current problem, searching for biomarkers to predict responses to pro-oxidative therapy an urgent task in determining the optimal approach to cancer eradication.

Therefore, constructing a risk prediction model for ESCC management by screening prognosis-associated genes holds profound value. The Cancer Genome Atlas (TCGA), a project under the joint supervision of the National Human Genome Research Institute, is the largest database of cancer gene information and comprehensively represents cancer types while displaying a multitude of omics data [16]. Our study identified genes associated with ESCC prognosis and immune-related OS from TCGA and performed validation using the GEO database, providing novel insights into the prognosis of ESCC.

## Methods

### Data processing and differentially expressed gene screening (DEGs)

We downloaded RNA sequencing of 162 ESCC tissues and 11 paracancerous tissues and the clinical data of ESCC cases in TCGA (https://portal.gdc.cancer.gov/). We also searched OS-related genes (OSRGs) from the msigdb.org website, and found the M5936 genome (HALLMARK_OXIDATIVE_PHOSPHORYLATION) by searching, which had 200 OSRGs in total (Suppl. Table S1). We adopted the ‘limma’ package for analysing the differential expressed genes and used Log2 multiple change (FC)≥1&&*P*<0.05 as threshold for screening DEGs. In addition, we constructed one validation cohort (GSE53625), containing RNA sequencing of 358 patients and the corresponding survival data in GEO (https://www.ncbi.nlm.nih.gov/geo/). We removed data loss and samples of NX, TX, and MX during the TCGA clinical data analysis.

### Analysis of gene ontology (GO) and kyoto encyclopedia of gene and genome from the perspective of gene and genome

Through “clusterProfiler”, “ggplot2”, as well as “enrichplot” software packages in R, the GO enrichment and the signal path of KEGG were noticed to explore the effects of those identified DEGS. *p* < 0.05 indicates significant difference.

### Establishment of LASSO prognostic model

LASSO regression pipeline was adopted for filtering overlapping differential expressed genes to narrow the range of target genes. With univariate Cox analysis, survival-associated genes were screened out. The ‘glmnet’ package as well as ‘survival’ package was adopted for analysing the risk features of the Cox regression model. With the help analysis, the risk formula was Risk score (RS) = $\sum_{i}^{n}Xi\times Yi$ (X: each gene’s coefficient, Y: each gene’s expression). Based on the median score, STAD patients from TCGA were assigned to low-risk (LR) and high-risk (HR) groups. A K-M survival curve was adopted for analysing and comparing the two groups’ overall survival, and time-associated ROC was adopted for evaluating the forecast value of gene markers. Finally, an overall survival risk features-based nomogram was adopted as one prognostic prediction tool for ESCC, to evaluate the prognosis discrimination ability as well as the accuracy of the model.

### Enrichment analysis of gene sets

Through GSEA software (4.0.3), the enrichment analysis on gene sets in HR and LR groups was carried out for an exploration of functional enrichment pathways of these chosen genes and inferring their effects. Genomes meet the standard value (*p*), FDR (Q) and other statistical indicators that are less than 0.05, which were considered statistically significant.

### Immune cell infiltration assessment and tumor microenvironment score generation

Through the CIBERSORT, We calculated the distribution and importance of immune cell subtypes using TCGA cohort, and used the abundance of six immune cell types of TIMER. For exploring the immune cell infiltration, the xCell and ESTIMATE algorithms were used for generating estimated scores, immune scores, as well as matrix scores for more deeper analysis of tumor microenvironment. Later analysis was performed with the results with *p* < 0.05. With Spearman’s correlation, the association of RS with tumor microenvironment score was determined. In addition, the 2-way ANOVA analysis was adopted for determining association of RS with immune infiltration subtype. Finally, we utilized ImmuCellAI to evaluate the infiltration of 24 immune cells, and Spearman’s correlation analysis was used to estimate the relationship between gene expression and immune cell infiltration, applying |r| > 0.2, *p* < 0.05, and FDR < 0.05 as the significance thresholds.

### Collection of clinical samples

30 patients with esophageal cancer who received surgical treatment at our hospital from January 2019 to January 2022 were collected, and both tumor tissues and corresponding adjacent non-tumor tissues were obtained, and record the patient’s follow-up data. This study received approval from our hospital’s Medical Ethics Committee. All participants provided verbal informed consents.

### qRT-PCR assay

Tissues were obtained and total RNA was extracted using GenElute Total RNA Purification Kit (Sigma-Aldrich; Merck KGaA, Beijing, China). Then, the concentration and quality of total RNA were analyzed using NanoDrop 2000 (Thermo Fisher Scientific, Inc., Shanghai, China). Reverse transcription was performed using RNA with (OD260)/OD280 (260 Nm) close to 2.0. The SuperScript® Vilo cDNA Synthesis Kit (Invitrogen, CA, USA) was used for reverse transcription with 2 μg of total RNA. The levels of MPC1, COX6C, CYB5R3, CASP7, and CYCS were quantified using the SYBR green I Master Mix Kit (Invitrogen; Thermo Fisher Scientific, Inc., Shanghai, China) through quantitative real-time PCR (qPCR). The 7500 real-time PCR system (Applied Biosystems; Thermo Fisher Scientific, Inc., Shanghai, China) was used for qPCR according to the manufacturer’s instructions, with GAPDH serving as the endogenous control for mRNA qPCR was performed under thermal cycling conditions: initial denaturation at 95°C for 10 min, followed by 40 cycles of 95°C for 20 s, 60°C for 15 s, and 72°C for 20 s. Final gene expression was calculated using the 2^−ΔΔCT^ method. The primer sequences are detailed in Table 1.

**Table 1:** Primer sequences for 5 signature genes

Symbol		Sequence (5′ -> 3′)	Length	Tm	Location
MPC1	Forward primer	TTATCAGTGGGCGGATGACAT	21	61.0	149–169
	Reverse primer	GCTGTACCTTGTAGGCAAATCTC	23	60.9	223–201
COX6C	Forward primer	CCAAAACCTCGGATGCGTG	19	61.4	19–37
	Reverse primer	AAATCTGCGTATGCCTTCTTTCT	23	60.3	155–133
CYB5R3	Forward primer	AAAGTCCAACCCTATCATCAGGA	23	60.5	420–442
	Reverse primer	AAGCGTGCAGAATGTTTGTTC	21	60.0	629–609
CASP7	Forward primer	AGTGACAGGTATGGGCGTTC	20	61.6	240–259
	Reverse primer	CGGCATTTGTATGGTCCTCTT	21	60.1	403–383
CYCS	Forward primer	CTTTGGGCGGAAGACAGGTC	20	62.8	108–127
	Reverse primer	TTATTGGCGGCTGTGTAAGAG	21	60.1	161–141
GAPDH	Forward primer	GGAGCGAGATCCCTCCAAAAT	21	61.6	108–128
	Reverse primer	GGCTGTTGTCATACTTCTCATGG	23	60.9	304–282

## Results

### Identification of DEGs

**Figure 1:**
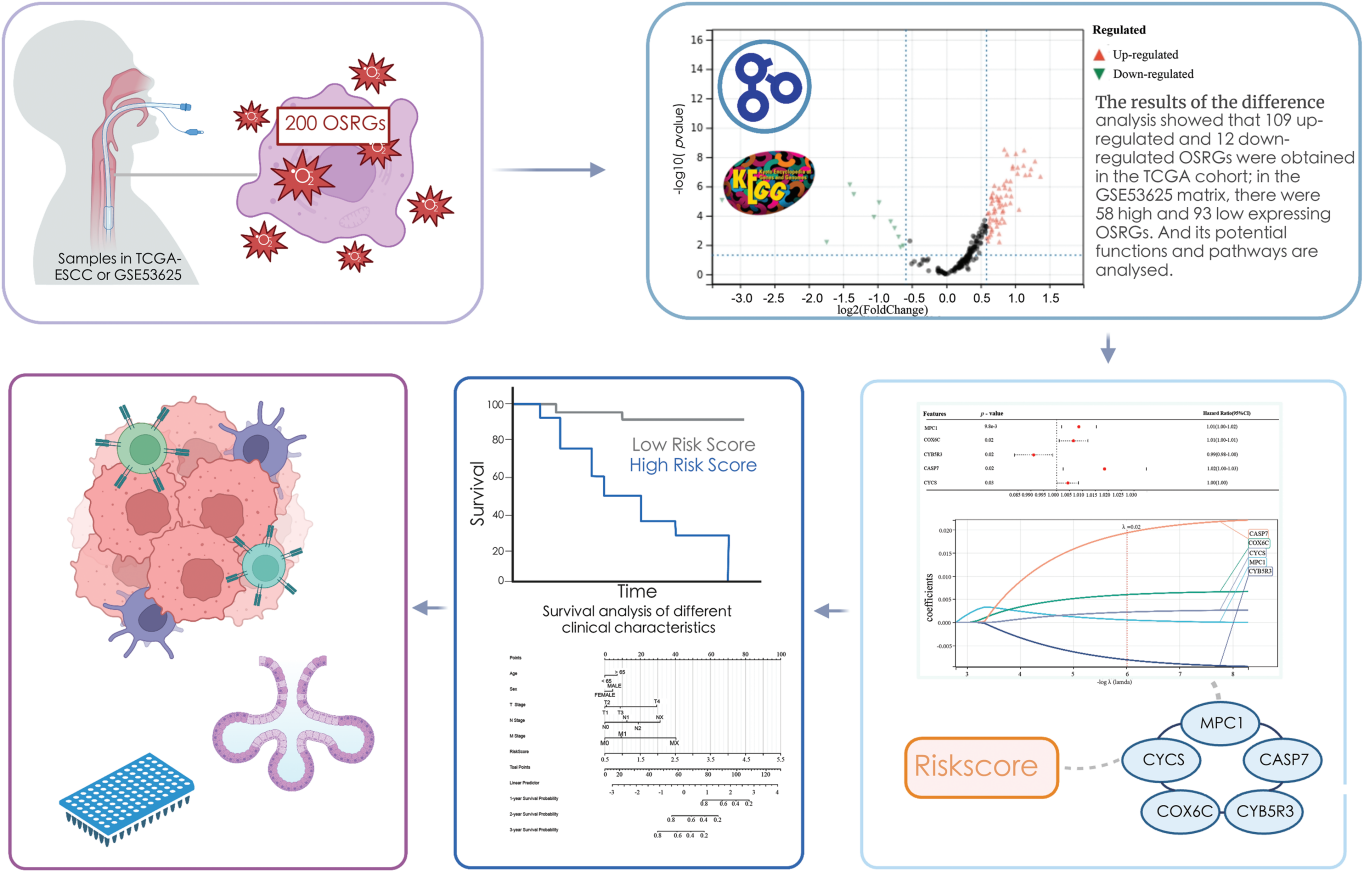
Design and roadmap of the study.

For the systematic representation of our research, we constructed a flow chart (Fig. 1). From the TCGA RNA-seq data set, we acquired 173 samples. In addition, OSRGs were searched from http://msigdb.org and the M5936 genome (HALLMARK_OXIDATIVE_PHOSPHORYLATION) which comprises 200 OSRGs.

The current study was based on TCGA’s ESCC dataset and the GSE53625 dataset to screen for prognostic value-based signatures in 200 genes associated with oxidative stress and to construct risk models. Various analyses were performed to validate the validity and convenience of the model, as well as the immunological and clinical significance of the model for ESCC.

**Figure 2:**
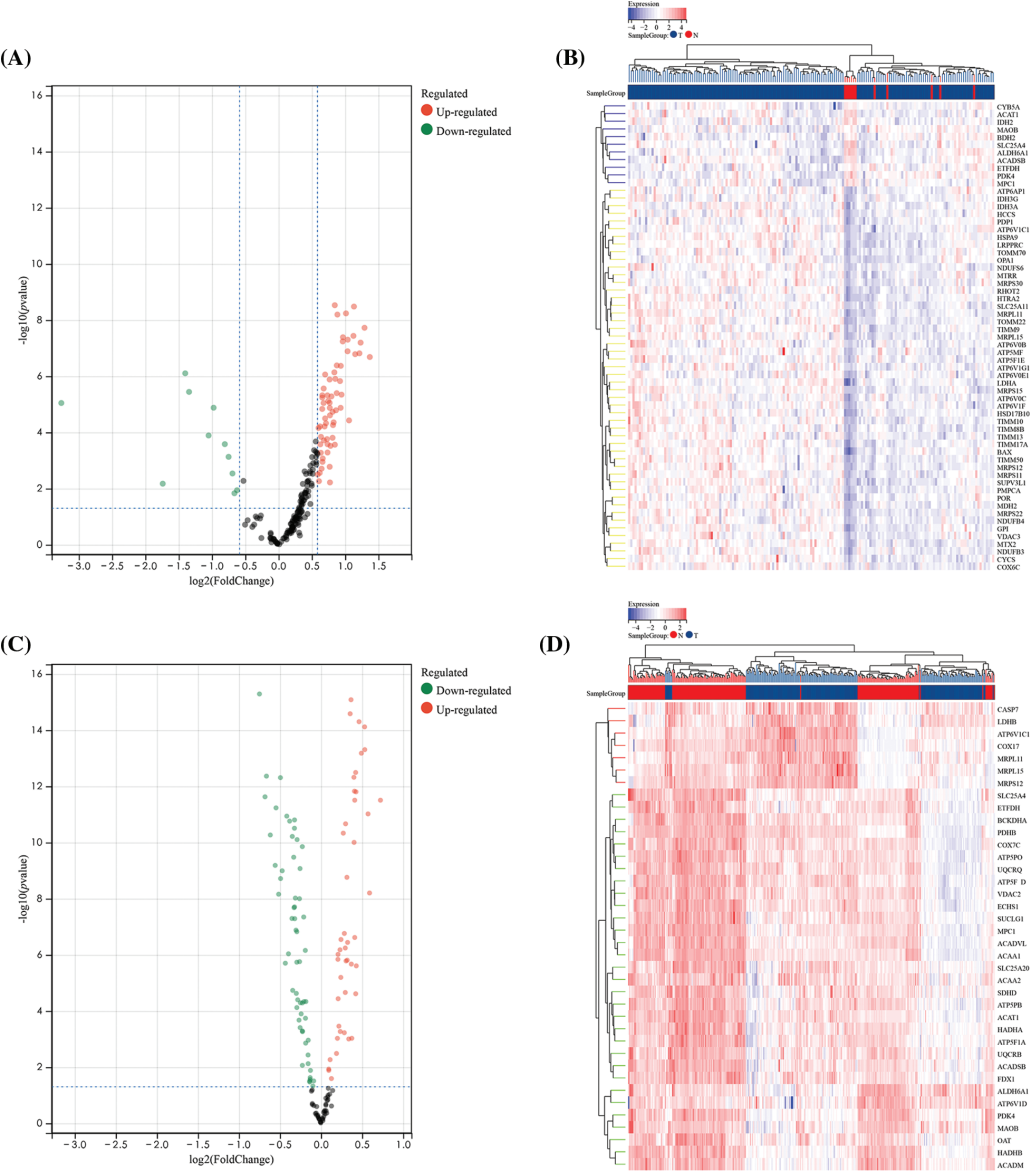
Differential analysis of 200 oxidative stress-related genes (OSRGs) in TCGA and GSE53625 datasets. (A and B) Volcano plot and heatmap of differentially expressed genes (DEGs) in the TCGA dataset. (C and D) Volcano plot and heatmap of DEGs in the GSE53625 dataset.

**Figure 3:**
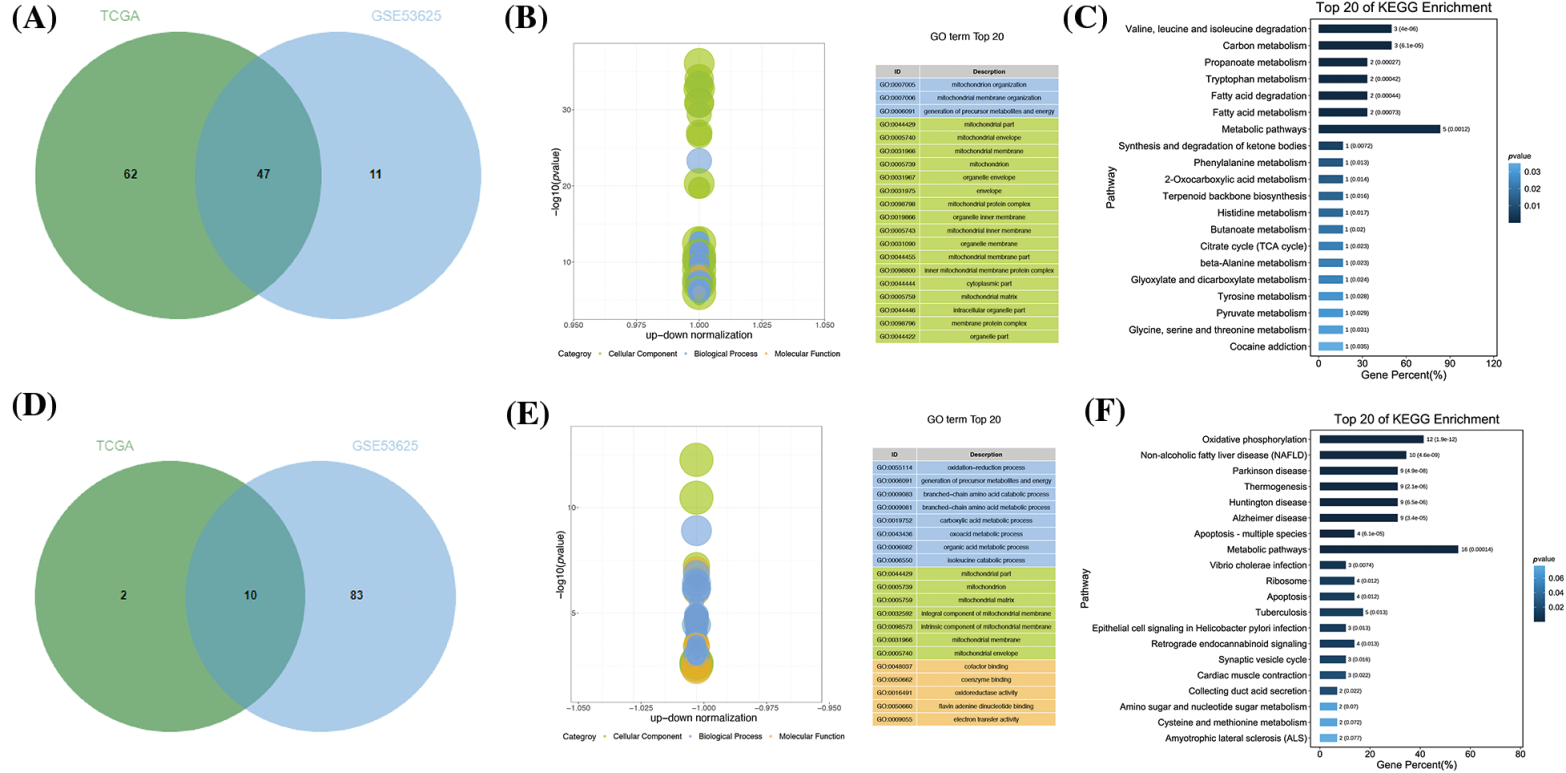
Different expressions of common DEGs in TCGA and GSE53625 datasets. (A) Venn diagram depicting the analysis of DEGs with consistently low expression in both TCGA and GSE53625 datasets. (B) Enrichment analysis of biological pathways associated with 47 identified genes. (C) Analysis of related pathways for the same set of genes. (D) Venn diagram illustrating DEGs with consistently high expression in both TCGA and GSE53625 datasets. (E) Enrichment analysis of biological pathways associated with 10 identified genes. (F) Analysis of related pathways for this latter group of genes.

The TCGA matrix containing 173 samples and 200 OSRGs was obtained by integration, including 162 cancer samples and 11 paracancerous samples. Moreover, based on 200 OSRGs, the GSE53625 matrix was integrated. The microarray contained 358 samples, including 179 cancer samples and 179 paracancerous samples. The differences were analyzed by limma package in R. There were 77 high-expression genes and 12 low-expression genes in the TCGA matrix (Suppl. Table S2), and 17 high-expression genes and 41 low-expression genes in the GSE53625 matrix (Suppl. Table S3). We obtained the intersection of these datasets using a Venn diagram Totally 10 over-expressed genes and 47 under-expressed genes (Fig. 2) were acquired. We attempted to further study these potential molecular mechanisms that determine the functional effects of differential OSRGs on the progress of ESCC through GO and KEGG analysis. The enrichment analysis revealed that DEOSRGs are mainly enriched in Oxidative phosphorylation, Apoptosis—multiple species, and Metabolic pathways related (Fig. 3).

### Association of OSRGs with the prognosis of ESCC

**Figure 4:**
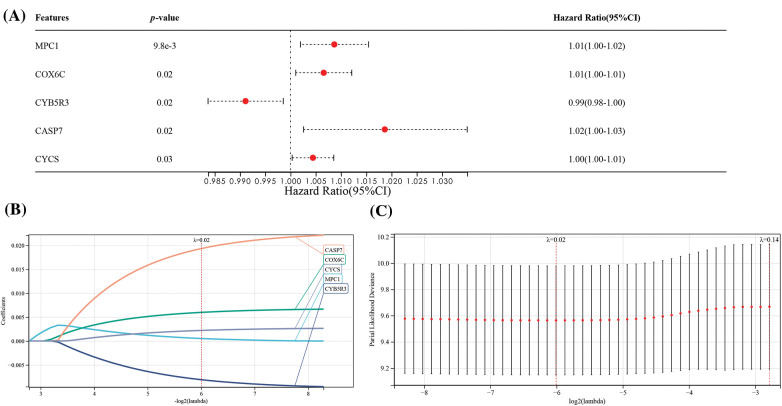
Construction of prognostic features of OSRGs in the TCGA dataset. (A) Univariate analysis on the association of five OSRGs with the prognosis of ESCC. (B and C) LASSO analysis for confirming the number of factors and building a prognostic prediction model.

Samples with missing clinical data and those with survival less than 30d from the TCGA matrix were deleted, and a matrix containing 57 related genes and 127 clinical samples was obtained. Then MPC1, COX6C, CYB5R3, CASP7, and CYCS were screened out via univariate Cox and LASSO regression analyses (Figs. 4A–4C). Based on 5 strong prognostic markers, risk features were established, and the RS was counted according to each marker’s coefficient acquired by LASSO analysis:

RS = 0.00051 * MPC1 + 0.00598 * COX6C − 0.0080 * CYB5R3 + 0.01933 * CASP7 + 0.00220 * CYCS.

**Figure 5:**
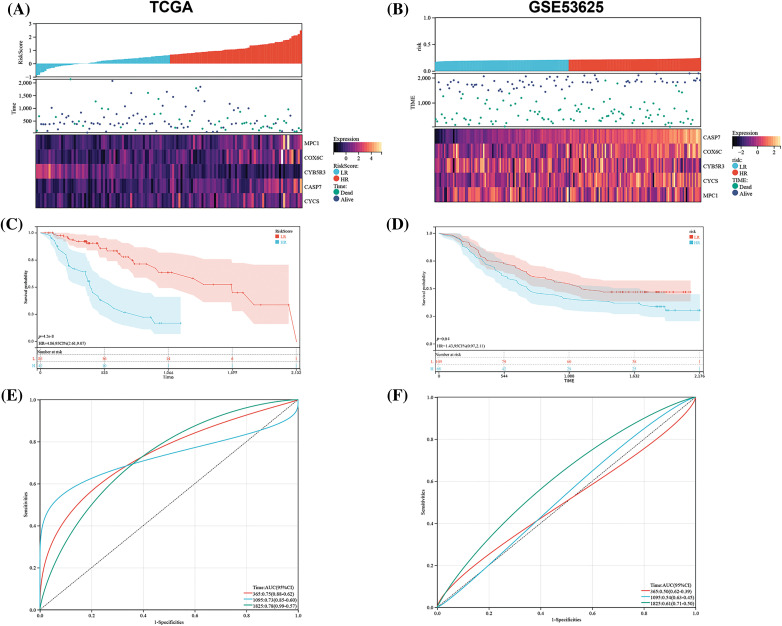
Prognostic significance of OSRGs in ESCC. In the TCGA and GSE53625 matrices, (A and B) the relationship between the OSRG prognostic model and gene expression in the model; (C and D) K-M Survival curves in HR and LR patients and (E and F) time-dependent ROC (T-D ROC). Note: LR: Low-risk; HR: High-risk.

Based on the best cutoff about RS, TCGA-ESCC tumor samples were grouped: HR/LR groups (Fig. 5A). We also discovered a notably shorter total survival time in the former than in the latter (Fig. 5C). In terms of evaluating the sensitivity and specificity, the AUC values acquired from the time-dependent ROC (T-D ROC) were acquired, which revealed that the AUC values of RS in forecasting patients’ survival within 1 to 5 years were no less than 0.73, which showed that the survival rate of patients can be analyzed based on this model (Fig. 5E). These results underscore the significant value of our model in forecasting the prognosis of ESCC patients.

### Verification of the risk model using external data

For further verifying the generalization of the model, we used an external data set (GSE53625) verification. By the RS classifier and K-M analysis, the difference in survival between groups was assessed, and the stability of the hypoxia risk model was verified. The external data were found to be similar to TCGA-ESCC (Fig. 5B). The results showed a strong ability of the model in forecasting patients’ prognosis with ESCC within 1, 3, and 5 years. The external validation cohort also revealed a poor survival rate in the HR group and a better one in the LR group (Fig. 5D, *p* = 0.010). We analyzed the ROC curve to further verify the accuracy of the RS model (Fig. 5F), and revealed that our risk model had certain generalization.

### Associations of RS with prognostic factors and clinical data

**Figure 6:**
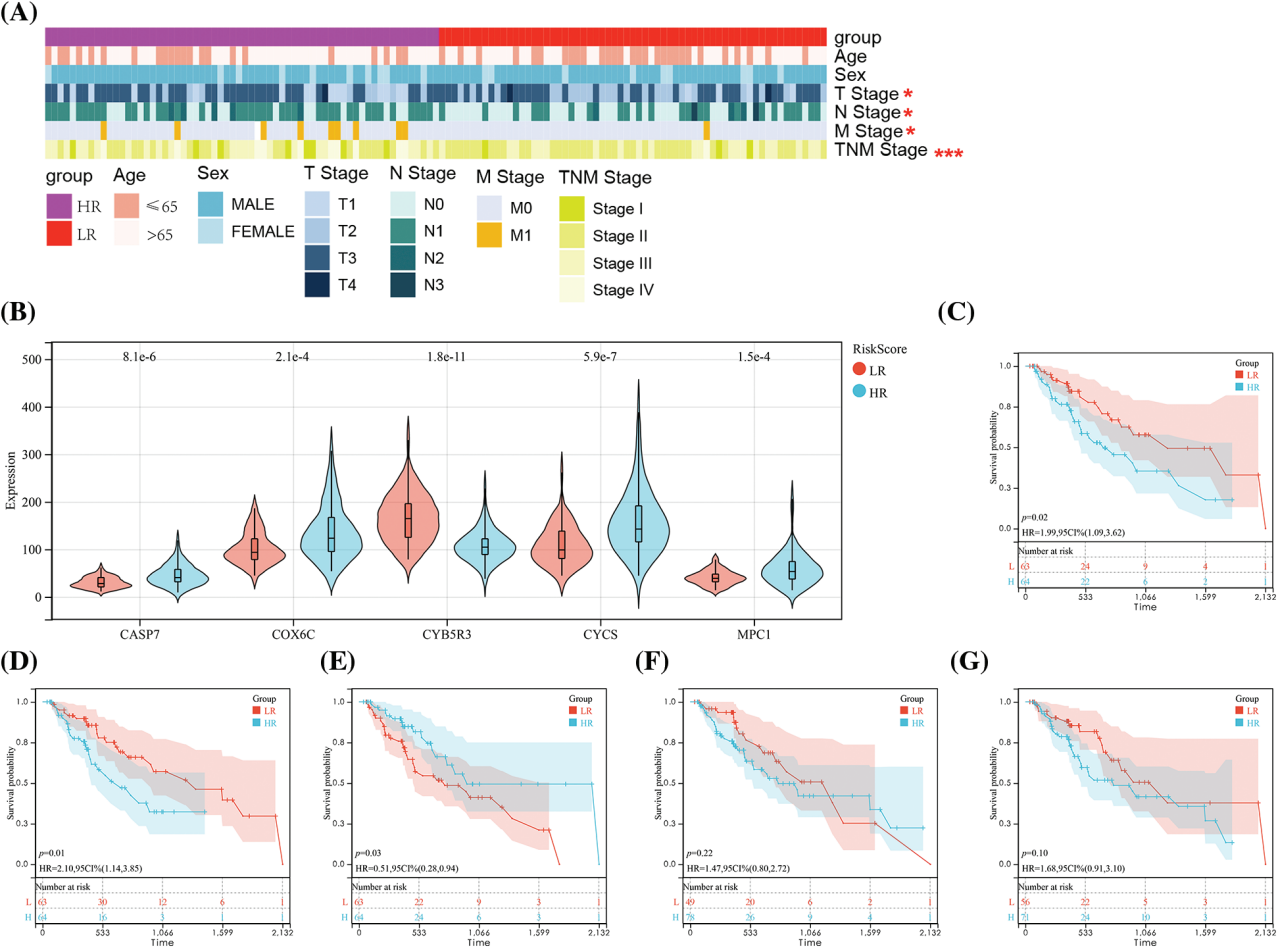
Correlation of risk score (RS) with clinical data of ESCC. (A) Comparison of clinical data between the HR and LR groups. (B) Expression of five OSRGs in the HR and LR groups. and K-M survival curve analysis of the value of (C) MPC1, (D) COX6C, (E) CYB5R3, (F) CASP7, (G) CYCS gene in patients’ prognosis. Notes: *p < 0.05, ***p < 0.001.

An analysis of the association of RS with ESCC patients’ clinical data showed notable difference between the LR and HR groups in T, N, and M staging and TNM staging (Fig. 6A). Specifically, greatly more patients with high staging were in T3–T4, N1–N3, M1 stage and III–IV stage than those with low staging. In addition, the HR group showed notably higher levels of MPC1, COX6C, CASP7 and CYCS and lower CYB5R3 expression than the LR group (Fig. 6B). The results implied that MPC1, COX6C, CASP7 and CYCSB were the risk factors for ESCC patients’ prognosis, while CYB5R3 was the protective factor of ESCC, which was verified by K-M survival curve (Figs. 6C–6G).

### Performance and verification of the nomogram

**Figure 7:**
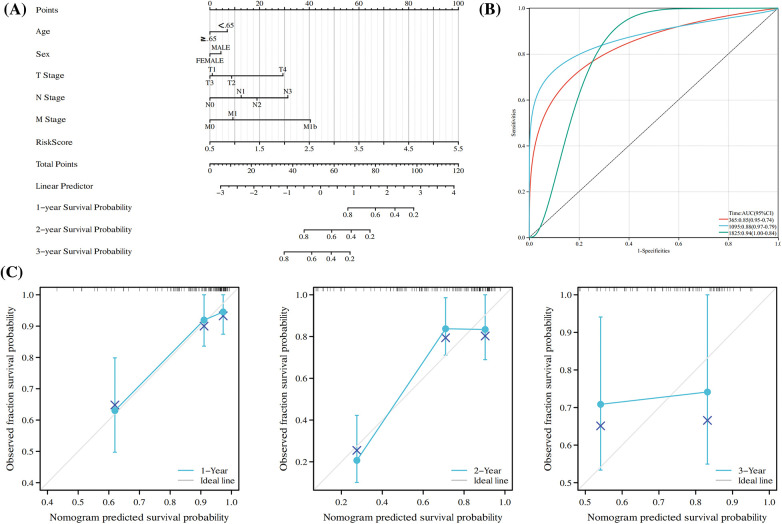
Construction of a Nomogram Based on the expression of five hub OSRGs. (A) Nomogram of RS and other clinical factors for the ESCC patients’ 1-, 2-, and 3-year overall survival in the TCGA cohort. (B) T-D ROC analysis of the predictive efficiency of risk features in the TCGA cohort. (C) Calibration plot of the nomogram in the TCGA cohort.

According to the results of the models of five overall survival genes and clinical attributes, a nomogram was constructed for forecasting the overall survival of ESCC patients in 1, 2 and 3 years (Fig. 7A). We used time-associated ROC curve for assessing anaphase of cure of the nomogram and found its AUC in forecasting the patients’ overall survival in 1, 3, and 5 years were 0.85, 0.88 and 0.94, respectively (Fig. 7B), suggesting the high accuracy and convenience of the model. From the standardization’ diagram, favorable alignment was found in TCGA (Fig. 7C). The calibration curve and ROC curve revealed the great potential of nomogram in clinical application.

### Association of molecular features with risk factors

Based on the previous results, the enrichment analysis of OSRGs pathways in TCGA showed enrichment of oxidative phosphorylation, apoptosis-multiple species and metabolic pathways.

**Figure 8:**
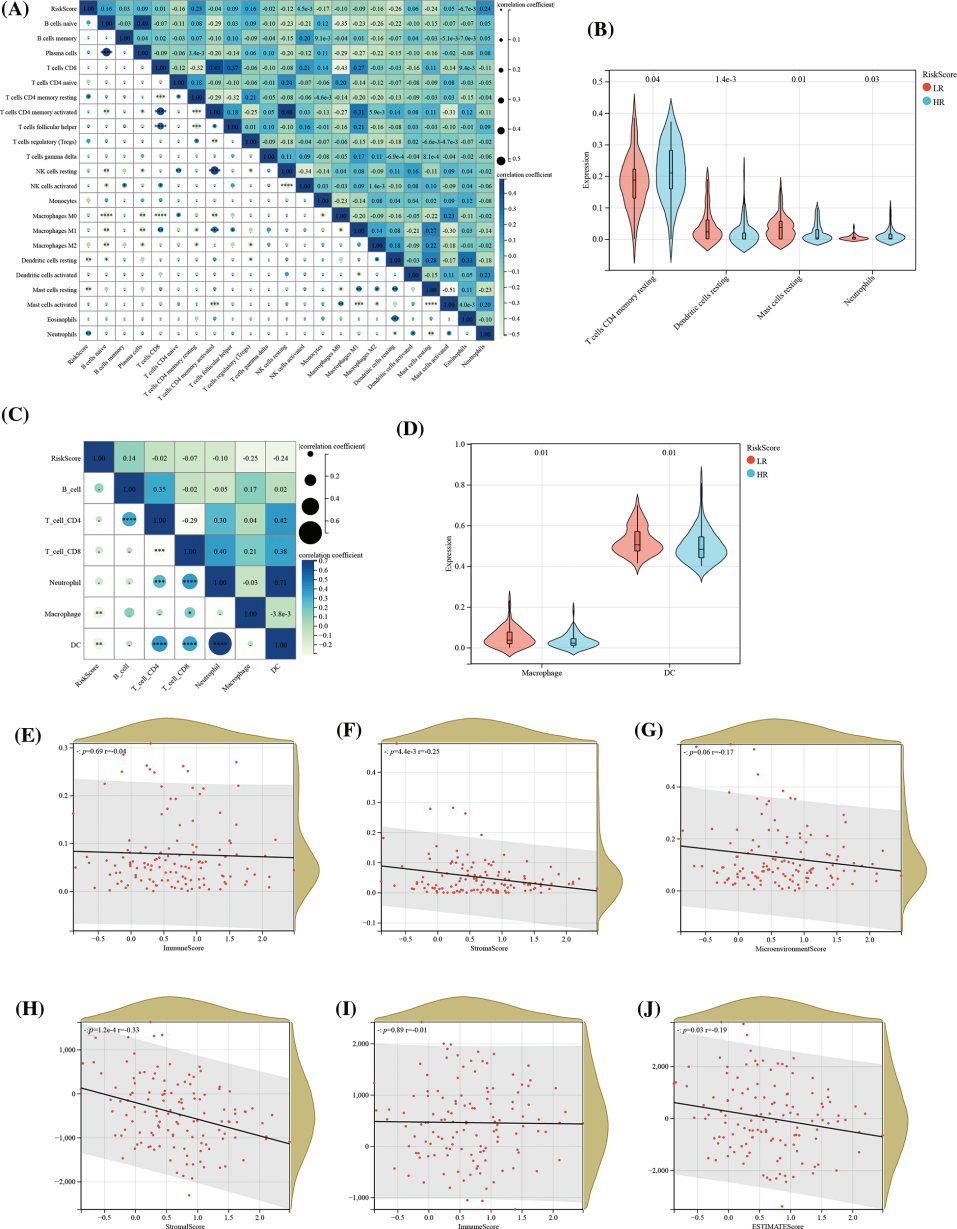
Association of RS with tumor immune infiltration. (A) Assessment of the correlation between the abundance of 22 immune cell types and RS, utilizing the CIBERSORT algorithm; (B) Comparative analysis of the abundance of 4 immune cell types across different risk groups; (C) Correlation determination between the abundance of 6 immune cell types and RS, implemented through the TIMER algorithm; (D) Comparative analysis of macrophage and dendritic cell abundance across different risk groups; Analysis of the association between RS and (E) immune score, (F) stroma score, and (G) microenvironment score, according to the xCell algorithm; as well as the association of RS with (H) immune score, (I) stroma score, and (J) ESTIMATE score in the context of the ESTIMATE algorithm.

**Figure 9:**
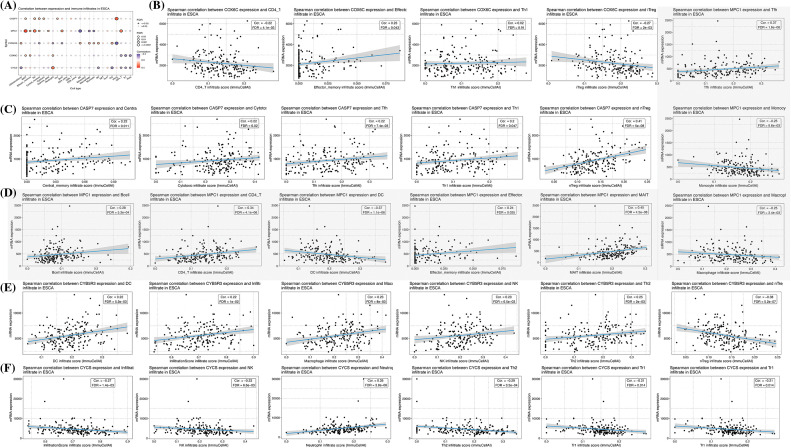
Association between gene expression and 24 immune cells. (A) Summary of correlations between five gene expressions and 24 immune cell types. Immune cells associated with (B) COX6C; (C) CASP7; (D) MPC1 (grey part); (E) CYB5R3; (F) CYCS expression.

Consequently, we analyzed the infiltration of immune cells in different groups. With immune cells in different groups. With the ESTIMATE, xCell, CIBERSORT, and TIMER algorithms, we calculated the levels of ESCC immune cells and immune score. The results revealed associations of mast cell-dendritic cell resting, memory T cell (CD4) resting as well as neutrophils in 22 immune cells with RS, suggesting the expression redundancy of the 4 cell types in various groups and the strong relationship of these different kinds of cells with RS (Figs. 8A and 8B). Through the TIMER algorithm, we found a negative association of RS with macrophage cells and DC cells, and an increase of the two kinds of cells in the LR group, which confirmed the role of OS-associated mRNA in tumor regulation: immune infiltration (Figs. 8C and 8D). Finally, the correlation analysis revealed negative associations of RS with stroma score and ESTIMATE score of CIBERSOR (Figs. 9A–9F). These results suggest that the features of OSRGs may substantially suppress or intensify the expression of non-common types of immune cells, which might reduce the therapeutic effect of immunotherapy.

### Analysis of biological function and its pathway

**Figure 10:**
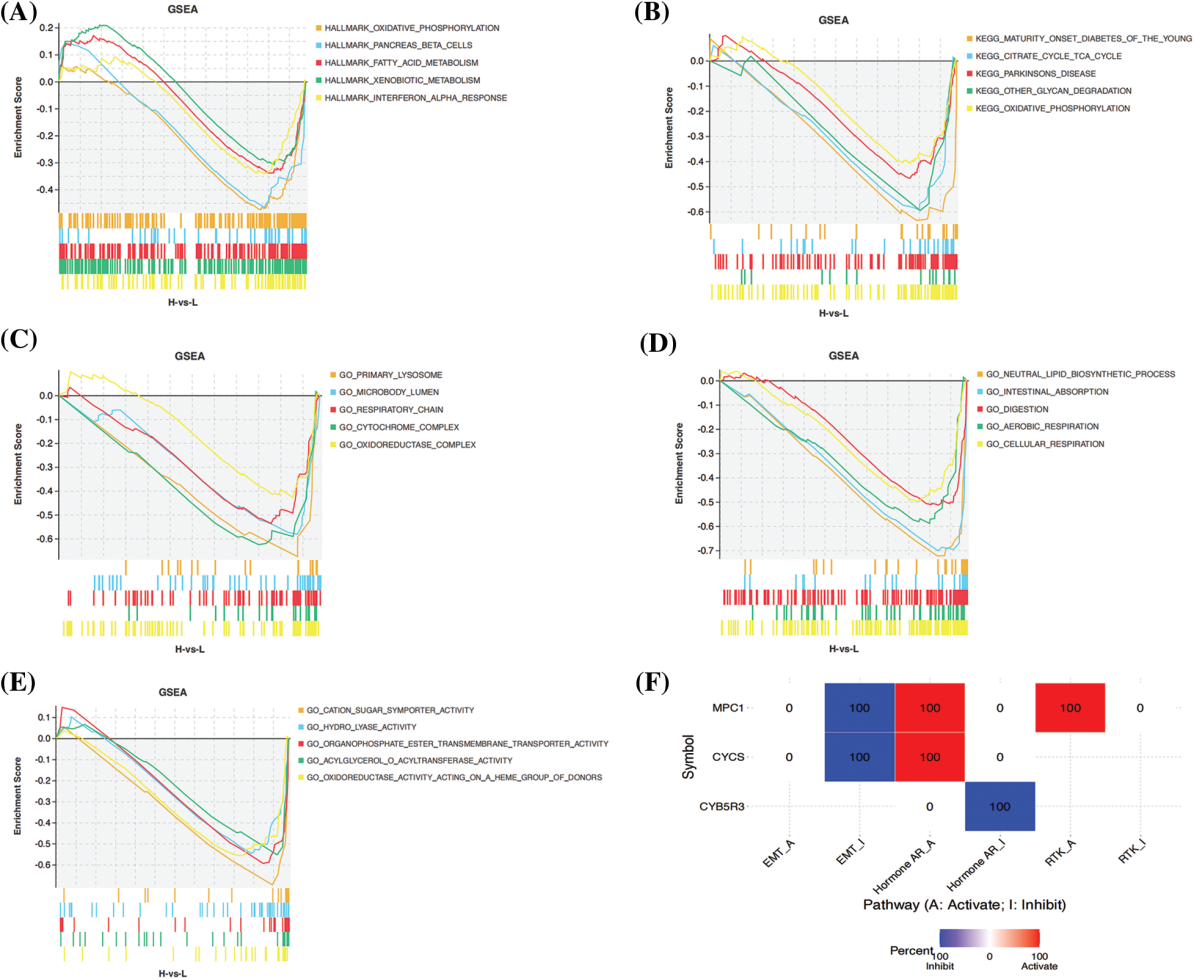
Gene set enrichment analysis of biological functions and pathways. (A) Hallmark, (B) KEGG and (C–E) other GOgene set. (F) Differences between gene expression and activity of common cancer-related pathways.

Finally, we carried out GSEA enrichment analysis. According to HALLMARK enrichment analysis, the HR population was primarily enriched with oxidative phosphorylation, iconic islet β-cells, fatty acid metabolism, heterogeneous biological metabolism, and interferon α response pathway. KEGG enrichment analysis revealed that patients in the HR group were enriched with peroxisome and oxidation-phosphorylation pathway. GO functional enrichment revealed differences in oxidoreductase complex, oxidative phosphorylation and oxidoreductase activity between the HR and LR groups (Figs. 10A–10E). The results suggest the possible impact of disorders of these signaling pathways in ESCC patients (HR/LR) on their prognosis. In addition, correlations were calculated between the expression of five genes and the pathway activity scores of 10 common cancer-related pathways, taking into account previous studies. It was found that MPC1 and CYCS were significantly associated with the inhibition of EMT signaling and activation of Hormone receptors, and MPC1 and CYB5R3 were in turn associated with the activation of RTK signaling and inhibition of Hormone receptors, respectively (Fig. 10F).

### Expression levels of prognostic genes in clinical patients

**Figure 11:**
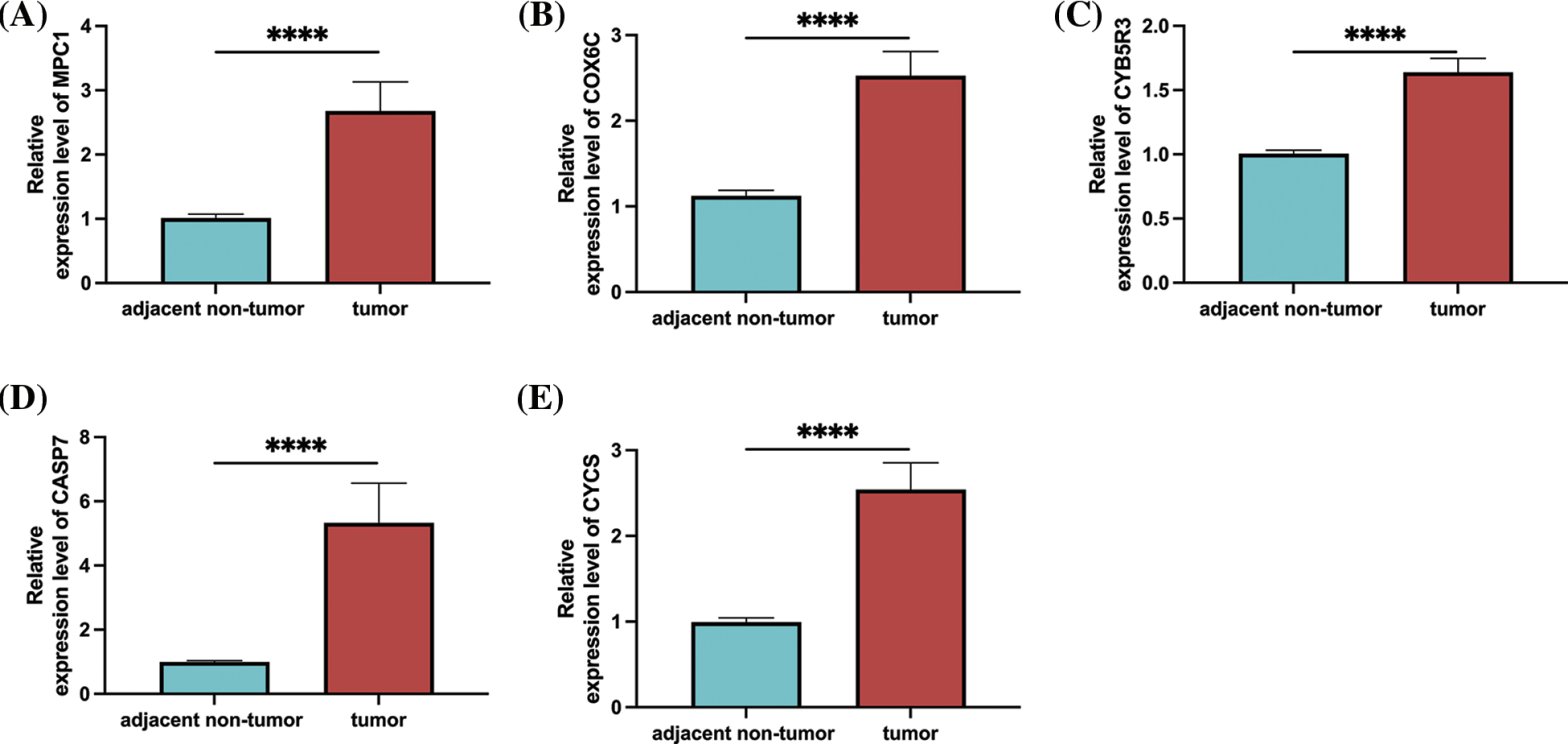
Prognostic gene expression in patients with esophageal cancer. (A) MPC1; (B) COX6C; (C) CYB5R3; (D) CASP7; (E) Relative expression levels of CYCS in tumor tissues and adjacent non-tumor tissues of patients with oesophageal cancer as determined by qRT-PCR. Note: ****p < 0.0001.

At the end of the study, to determine the expression of prognostic genes in esophageal cancer, we used qRT-PCR to detect the expression levels of these genes in tumor tissues and adjacent non-tumor tissues of esophageal cancer patients. Our results showed that the levels of MPC, COX6C, CYB5R3, CASP7, and CYCS were significantly upregulated in tumor tissues compared with adjacent non-tumor tissues in esophageal cancer patients (Figs. 11A–11E).

## Discussion

ESCC is one of the most fatal digestive tract tumors in adults, with serious malignant features in mortality and prognosis. Currently, the standard protocol of ESCC management involves total resection followed by chemotherapy and radiotherapy [17]. Despite the great progress in diagnostic techniques and multidimensional treatment methods of ESCC, the 5-year OSR of ESCC is still disappointing. As everyone knows, overall survival is related to the growth of other tumors [18]. However, the value of OSRGs on cancer patients’ survival and prognosis is still under investigation. Our study analyzed the impacts of OS molecular biomarkers on predicting the prognosis of ESCC and their influence on the immune microenvironment to provide the basis for treatment decisions.

In our study, based on TCGA and GSE53625 matrix, we identified 10 over-expressed and 47 under-expressed DEGs and systematically identified the related biological pathways of these genes. Enrichment analysis revealed the participation of those DEGs in oxidative phosphorylation and apoptosis and their strong associations with many metabolic pathways. The results suggest the strong association of DEGs with ESCC progress, which is of crucial significance for the comprehensive evaluation of the mechanism of these 57 OSRGs. Therefore, we screened prognosis-associated DEGs by LASSO/single factor Cox regression analysis and found 5 Hub OSRGs: MPC1, COX6C, CYB5R3, CASP7 and CYCS, which were intimately associated with the poor prognosis of ESCC patients. These genes have been verified to be associated with cancer in prior research. For example, Chai et al. [19] have revealed the association of MPC1 deletion with the poor prognosis of glioblastoma and temozolomide resistance. COX6C is differentially expressed in thyroid cancer and melanoma [20]. Overexpression of caspase 7 affects the proliferation as well as growth of breast cancer (BC) cells [21]. After analysis, CYCS reveals a significant threat to BC in the process of prognosis and metastasis [22]. Although the regulatory role of these genes has been explored in various tumors, few studies have systematically analyzed their specific prognostic value in ESCC.

We used the expression data from TCGA to construct a risk model with the five Hub OSRGs. Based on the median value, the patients were assigned to HR or LR groups for analyzing their clinical data.

According to the results, the former group was strongly bound up with adverse outcomes: In the HR group, the ratio of late-stage N, M and III–IV increased, and the overall survival time was shorter, which was in agreement with the significance of clinical features in predicting overall survival. Except for CASP7 and CYCS, K-M analysis showed that MPC1, COX6C and other genes had negative correlations with the overall survival of ESCC patients, while CYB5R3 had a positive correlation with them, suggesting the strong association of the overall expression of Hub OSRGs with ESCC’ prognosis. In addition, through the associations of RS and clinical features, we found that the HR score was strongly linked to the high grade or staging of tumor, suggesting a direct correlation of high RS with the unfavorable prognosis of ESCC. As an independent prognostic reference factor, RS showed strong accuracy in the T-D ROC test. To further understand the association of RS with the patient’s prognosis, we conducted a nomogram analysis, and the RS also presented a stable performance in the prediction of individual prognosis according to the nomogram. To generalize the RS, the external dataset GSE53625 was adopted for verification. According to the results of bioinformatics analysis, the risk features constructed with 5 OSRGs can be adopted for the overall survival prognosis, with a favorable prognostic value, which provides a new thinking direction for further expanding the molecular mechanism of ESCC.

The tumor microenvironment might affect tumor progression and recurrence and is thus becoming an increasingly hot topic [23]. According to research, the complex interaction between tumor cells and tumor microenvironment greatly affects tumor formation and takes a crucial part in immunotherapy and overall survival rate [24]. Reportedly, the heterogeneity of tumors depends on tumor cells and tumor microenvironment, and the tumor microenvironment is composed of immune cells and other cell types [25]. Moreover, during the development of tumors, the tumor cells will also induce the micro-environment of the immunosuppressive microenvironment to counteract anti-cancer immunity, which will lead to the immune escape of the cancer cells [26]. Typically, the cells and molecules are dynamic in the tumor microenvironment, and a significant portion of immunosuppressive cells congregate in the tumor microenvironment, affecting the tumor prognosis by regulating immune escape, tumor growth, and metastasis [27]. Our study found that 5 risk features of OSRGs construction were implicated in the immune pathway, and the number of four kinds of immune cells, including resting dendritic cells, resting CD4 memory T cells, mast cells sleeping, as well as neutrophils, was greatly different between the HR and LR groups, indicating a strong role of OSRGs in regulating tumor immune infiltration. Tumor-associated resting CD4 memory T cells is the main immune component of many tumors, which can promote tumor progression [28]. As a class of anti-prototype and proliferating cells, dendritic cells infiltration into tumors is considered the host’s immune response to tumors [29]. Prior research has revealed that the increase of tumor-associated mast cells and neutrophils is bound up with the unfavorable prognosis of ESCC patients because of their role in immune invasion [30]. In a word, the four survival-associated cells found above may take a crucial part in immune infiltration and ESCC immunotherapy, which verifies the effectiveness of cell-based gene analysis related to OS. At the end of the study, we assigned TCGA-ESCC samples to HR or LR groups based on RS. Through GSEA analysis, HR patients were found to be significantly rich in OS-related signal pathways, which offered a new idea for treatment. In addition, we found by qRT-PCR that the levels of MPC1, COX6C, CYB5R3, CASP7, and CYCS in tumor tissues of esophageal cancer patients were significantly higher than adjacent non-tumor tissues, suggesting that these genes may have some value in clinical diagnosis and prognosis. To further determine the significance of the above genes, we collected patient survival data, obtained each patient and obtained a risk score through the risk model, showing that the survival rate in the high-risk group was significantly higher than the low-risk group, and this model showed in time-dependent ROC, The risk model has a reference value in predicting 1-year and 3-year survival. In the previous study of Liu et al. [31], the prognostic model of oxidative stress in esophageal cancer was also established through the TCGA database, but compared with their study, we also established a nomogram, and the expression of genes in esophageal cancer tissue was detected by qRT-PCR, which is more conducive to the establishment of the clinical prediction model.

In this study, OSRGs predicted patients’ prognosis for ESCC, and a valid scoring model containing five Hub OSRGs was established. Further, we found that these five OSRGs models were associated with the tumor microenvironment and the immune infiltration level, providing new ideas for future clinical treatment. Our study provides an important tool for personalized treatment decision-making for ESCC patients. The discovery of OSRGs could help clinicians to better predict patient outcomes and provide patients with more personalized and effective treatment options. Moreover, through the association analysis with the tumor microenvironment and the immune infiltration level, we can gain a deeper understanding of the mechanisms of tumor growth and metastasis and provide more scientific and precise guidance for future treatment strategies. In future studies, we will continue to explore the relationship of these OSRGs with ESCC development and further validate the function and interaction of these genes. We will also verify the feasibility and effectiveness of these genes in the treatment of ESCC through large-scale clinical experiments, and provide more scientific basis for the individualized treatment of ESCC.

However, this study still has certain limitations. Firstly, although we detected the expression in tumor tissues of esophageal cancer patients, further research is needed to investigate whether these genes have the same expression and regulatory effects in other esophageal cancer models. Secondly, in this study, we only collected cancerous and adjacent tissues. Whether OSRGs have diagnostic value in ESCC requires more normal samples to verify our conclusions. Although our research results have a certain degree of credibility, they still need more to be confirmed with our clinical data. Therefore, we hope to conduct various experiments in future research to comprehensively analyze the relationship between these genes and ESCC.

To sum up, we have constructed OSRGs of ESCC for forecasting patients’ prognosis, and we have constructed one effective nomogram containing 5 Hub OSRGs. The most important thing is that our research has revealed the possible association of 5 OSRGs models with the immune infiltration level and tumor micro-environment, which provides novel ideas for clinical therapy.

## Supplementary Materials

Table S1 200 genes associated with oxidative stress

Table S2 Results of differential analysis of oxidative stress genes in the TCGA dataset

Table S3 Results of differential analysis of oxidative stress genes in the GEO dataset

## Data Availability

All data for this study can be obtained by contacting the authors.

## Supplementary Materials

The supplementary material is available online at https://doi.org/10.32604/or.2023.030969.
