# Supplementary material for: A prognosis model for predicting immunotherapy response of esophageal cancer based on oxidative stress-related signatures
Source: Oncol Res. 2023 Nov 15;32(1):199–212. doi: 10.32604/or.2023.030969 (PMC10774069; doi:10.32604/or.2023.030969)
Supplement: Table S1 [file OncolRes-32-30969-s001.docx]

Table S1 200 genes associated with oxidative stress

| Gene | | | | | | | |
| --- | --- | --- | --- | --- | --- | --- | --- |
| ABCB7 | ACAA1 | ACAA2 | ACADM | ACADSB | ACADVL | ACAT1 | ACO2 |
| AFG3L2 | AIFM1 | ALAS1 | ALDH6A1 | ATP1B1 | ATP5F1A | ATP5F1B | ATP5F1C |
| ATP5F1D | ATP5F1E | ATP5MC1 | ATP5MC2 | ATP5MC3 | ATP5ME | ATP5MF | ATP5MG |
| ATP5PB | ATP5PD | ATP5PF | ATP5PO | ATP6AP1 | ATP6V0B | ATP6V0C | ATP6V0E1 |
| ATP6V1C1 | ATP6V1D | ATP6V1E1 | ATP6V1F | ATP6V1G1 | ATP6V1H | BAX | BCKDHA |
| BDH2 | CASP7 | COX10 | COX11 | COX15 | COX17 | COX4I1 | COX5A |
| COX5B | COX6A1 | COX6B1 | COX6C | COX7A2 | COX7A2L | COX7B | COX7C |
| COX8A | CPT1A | CS | CYB5A | CYB5R3 | CYC1 | CYCS | DECR1 |
| DLAT | DLD | DLST | ECH1 | ECHS1 | ECI1 | ETFA | ETFB |
| ETFDH | FDX1 | FH | FXN | GLUD1 | GOT2 | GPI | GPX4 |
| GRPEL1 | HADHA | HADHB | HCCS | HSD17B10 | HSPA9 | HTRA2 | IDH1 |
| IDH2 | IDH3A | IDH3B | IDH3G | IMMT | ISCA1 | ISCU | LDHA |
| LDHB | LRPPRC | MAOB | MDH1 | MDH2 | MFN2 | MGST3 | MPC1 |
| MRPL11 | MRPL15 | MRPL34 | MRPL35 | MRPS11 | MRPS12 | MRPS15 | MRPS22 |
| MRPS30 | MTRF1 | MTRR | MTX2 | NDUFA1 | NDUFA2 | NDUFA3 | NDUFA4 |
| NDUFA5 | NDUFA6 | NDUFA7 | NDUFA8 | NDUFA9 | NDUFAB1 | NDUFB1 | NDUFB2 |
| NDUFB3 | NDUFB4 | NDUFB5 | NDUFB6 | NDUFB7 | NDUFB8 | NDUFC1 | NDUFC2 |
| NDUFS1 | NDUFS2 | NDUFS3 | NDUFS4 | NDUFS6 | NDUFS7 | NDUFS8 | NDUFV1 |
| NDUFV2 | NNT | NQO2 | OAT | OGDH | OPA1 | OXA1L | PDHA1 |
| PDHB | PDHX | PDK4 | PDP1 | PHB2 | PHYH | PMPCA | POLR2F |
| POR | PRDX3 | RETSAT | RHOT1 | RHOT2 | SDHA | SDHB | SDHC |
| SDHD | SLC25A11 | SLC25A12 | SLC25A20 | SLC25A3 | SLC25A4 | SLC25A5 | SLC25A6 |
| SUCLA2 | SUCLG1 | SUPV3L1 | SURF1 | TCIRG1 | TIMM10 | TIMM13 | TIMM17A |
| TIMM50 | TIMM8B | TIMM9 | TOMM22 | TOMM70 | UQCR10 | UQCR11 | UQCRB |
| UQCRC1 | UQCRC2 | UQCRFS1 | UQCRH | UQCRQ | VDAC1 | VDAC2 | VDAC3 |
