# Supplementary material for: A prognosis model for predicting immunotherapy response of esophageal cancer based on oxidative stress-related signatures
Source: Oncol Res. 2023 Nov 15;32(1):199–212. doi: 10.32604/or.2023.030969 (PMC10774069; doi:10.32604/or.2023.030969)
Supplement: Table S2 [file OncolRes-32-30969-s002.docx]

Table S2 Results of differential analysis of oxidative stress genes in the TCGA dataset

| Gene | logFC | AveExpr | *t* | *p* value | adj.*p*.Val | B |
| --- | --- | --- | --- | --- | --- | --- |
| BAX | 1.651574223 | 5.189395326 | 9.403130215 | 2.53E-17 | 5.06E-15 | 28.77690551 |
| LDHA | 1.85049205 | 8.67492514 | 7.11975569 | 2.52E-11 | 2.52E-09 | 15.36869655 |
| MTX2 | 0.844527449 | 5.520514775 | 6.247371917 | 2.95E-09 | 1.65E-07 | 10.76615651 |
| ATP6V1F | 1.132442614 | 7.786343007 | 6.226201523 | 3.30E-09 | 1.65E-07 | 10.65905374 |
| TIMM50 | 1.012593003 | 4.385475447 | 6.11907952 | 5.77E-09 | 2.12E-07 | 10.12069362 |
| TIMM17A | 0.883842429 | 5.543220932 | 6.100259478 | 6.36E-09 | 2.12E-07 | 10.02673379 |
| ATP5MF | 1.291023563 | 7.215899905 | 5.888819175 | 1.88E-08 | 5.37E-07 | 8.984214438 |
| MRPL11 | 1.124676212 | 4.995768237 | 5.756804244 | 3.65E-08 | 9.07E-07 | 8.345809365 |
| HSD17B10 | 0.967504304 | 6.865644406 | 5.734596613 | 4.08E-08 | 9.07E-07 | 8.239383649 |
| MRPS12 | 1.039025319 | 5.137179691 | 5.694487154 | 4.98E-08 | 9.96E-07 | 8.047881621 |
| TOMM22 | 0.964093576 | 5.892833154 | 5.665581993 | 5.75E-08 | 1.05E-06 | 7.910448063 |
| POR | 1.232271631 | 5.702654527 | 5.645277394 | 6.36E-08 | 1.06E-06 | 7.814196044 |
| TIMM10 | 1.036646809 | 5.824719439 | 5.500567192 | 1.29E-07 | 1.98E-06 | 7.135201897 |
| GPI | 1.214127883 | 7.04691451 | 5.465737864 | 1.53E-07 | 2.18E-06 | 6.973632802 |
| NDUFS6 | 1.150587074 | 6.54424935 | 5.451710033 | 1.63E-07 | 2.18E-06 | 6.908764929 |
| PDP1 | 1.370445722 | 4.767040149 | 5.4031532 | 2.06E-07 | 2.58E-06 | 6.685143964 |
| HTRA2 | 0.874913744 | 3.786997703 | 5.258092233 | 4.11E-07 | 4.74E-06 | 6.025668217 |
| ATP6V1C1 | 0.934440021 | 5.133089108 | 5.250427162 | 4.26E-07 | 4.74E-06 | 5.991183205 |
| MRPS15 | 0.838117566 | 5.585097776 | 5.135825529 | 7.28E-07 | 7.66E-06 | 5.479992573 |
| SLC25A4 | -1.40155518 | 3.581923954 | -5.118987268 | 7.87E-07 | 7.87E-06 | 5.405584611 |
| NDUFB3 | 0.699475056 | 6.445993887 | 5.097656292 | 8.68E-07 | 8.27E-06 | 5.311583407 |
| MRPS22 | 0.852525073 | 4.153452996 | 5.019598383 | 1.24E-06 | 1.13E-05 | 4.970091958 |
| TIMM9 | 0.766867023 | 5.395101549 | 5.00831345 | 1.31E-06 | 1.14E-05 | 4.921048182 |
| MRPL15 | 0.923342468 | 6.463838448 | 4.980274946 | 1.49E-06 | 1.24E-05 | 4.799553135 |
| ATP5F1E | 0.677565242 | 6.356869126 | 4.844602154 | 2.73E-06 | 2.19E-05 | 4.218963544 |
| ALDH6A1 | -1.34401864 | 3.589663401 | -4.78218439 | 3.60E-06 | 2.77E-05 | 3.955971507 |
| MDH2 | 0.873397558 | 7.527398472 | 4.758689146 | 4.00E-06 | 2.96E-05 | 3.857656022 |
| MTRR | 0.945532826 | 4.027826945 | 4.730631769 | 4.52E-06 | 3.19E-05 | 3.740740413 |
| ATP6AP1 | 0.657861431 | 6.441876424 | 4.718664991 | 4.76E-06 | 3.19E-05 | 3.691037442 |
| IDH3G | 0.733891891 | 6.077649514 | 4.717709915 | 4.78E-06 | 3.19E-05 | 3.687074821 |
| NDUFB4 | 0.794371763 | 7.055111505 | 4.698306893 | 5.21E-06 | 3.36E-05 | 3.60670627 |
| ATP6V0E1 | 0.6568208 | 7.766602812 | 4.675731825 | 5.74E-06 | 3.59E-05 | 3.513523008 |
| HSPA9 | 0.745842803 | 7.13238548 | 4.612547235 | 7.55E-06 | 4.57E-05 | 3.254578951 |
| SUPV3L1 | 0.767066114 | 4.334634174 | 4.576053313 | 8.83E-06 | 4.99E-05 | 3.10627784 |
| MRPS11 | 0.676709808 | 3.327707554 | 4.572257911 | 8.97E-06 | 4.99E-05 | 3.090907582 |
| PDK4 | -3.262558065 | 3.458105133 | -4.572165912 | 8.97E-06 | 4.99E-05 | 3.090535139 |
| ACAT1 | -0.975436796 | 3.889551151 | -4.48124667 | 1.32E-05 | 6.86E-05 | 2.725360772 |
| IDH3A | 0.932158568 | 3.60112227 | 4.4790025 | 1.33E-05 | 6.86E-05 | 2.716420714 |
| PMPCA | 0.769552211 | 4.223214751 | 4.478166505 | 1.34E-05 | 6.86E-05 | 2.713091284 |
| ATP6V0B | 0.655651529 | 6.324002833 | 4.45759842 | 1.46E-05 | 7.29E-05 | 2.631332812 |
| LRPPRC | 0.862396205 | 6.069664809 | 4.431107855 | 1.63E-05 | 7.95E-05 | 2.526474735 |
| MRPS30 | 0.811422293 | 4.446593663 | 4.400155461 | 1.85E-05 | 8.82E-05 | 2.404588665 |
| TOMM70 | 0.771919581 | 5.796743596 | 4.330666133 | 2.47E-05 | 0.000114873 | 2.133451427 |
| HCCS | 0.699631094 | 4.979361279 | 4.269253577 | 3.17E-05 | 0.000144305 | 1.896732703 |
| VDAC3 | 1.058323333 | 6.130423075 | 4.228877662 | 3.74E-05 | 0.000166187 | 1.742597991 |
| CYCS | 0.827286632 | 6.907901277 | 4.189333977 | 4.38E-05 | 0.000190627 | 1.592799094 |
| ATP6V0C | 0.712228878 | 5.642010555 | 4.170781286 | 4.72E-05 | 0.000200963 | 1.522915061 |
| TIMM8B | 0.818139255 | 5.798528554 | 4.133076926 | 5.49E-05 | 0.000228682 | 1.381675608 |
| ATP6V1G1 | 0.623129948 | 7.547846192 | 4.105973805 | 6.11E-05 | 0.000245066 | 1.280800507 |
| COX6C | 0.691083781 | 6.741857048 | 4.105311228 | 6.13E-05 | 0.000245066 | 1.278341315 |
| RHOT2 | 0.600708794 | 4.996038573 | 4.074243623 | 6.92E-05 | 0.000271558 | 1.163400413 |
| ACADSB | -1.050524282 | 3.362606695 | -3.914756122 | 0.000128439 | 0.000493994 | 0.584795205 |
| SLC25A11 | 0.637453354 | 5.472442701 | 3.885591436 | 0.000143515 | 0.000541565 | 0.481081995 |
| OPA1 | 0.810953716 | 5.491757071 | 3.8646975 | 0.000155333 | 0.000575307 | 0.407182215 |
| TIMM13 | 0.728111355 | 5.713918184 | 3.836020568 | 0.000173065 | 0.000629326 | 0.306302469 |
| IDH3B | 0.680703597 | 6.181679611 | 3.807397209 | 0.000192666 | 0.000688092 | 0.206245241 |
| ATP5PD | 0.558599496 | 5.976031709 | 3.784438288 | 0.00020989 | 0.000736457 | 0.126448299 |
| ETFDH | -0.806967801 | 3.844696238 | -3.724969569 | 0.000261537 | 0.000886451 | -0.078333102 |
| PHB2 | 0.73513177 | 7.576420371 | 3.722847703 | 0.000263585 | 0.000886451 | -0.085588664 |
| VDAC1 | 0.62730427 | 7.647347791 | 3.720432409 | 0.000265935 | 0.000886451 | -0.093843283 |
| NQO2 | 0.845477202 | 2.817185649 | 3.712490732 | 0.000273803 | 0.000897714 | -0.120952851 |
| GPX4 | 0.780186426 | 7.686252484 | 3.6859407 | 0.000301731 | 0.000973327 | -0.211223705 |
| ATP6V1E1 | 0.534098613 | 6.091822243 | 3.587559392 | 0.000430472 | 0.001366579 | -0.540866605 |
| NDUFAB1 | 0.558810707 | 6.629824668 | 3.556699245 | 0.000480515 | 0.00150161 | -0.642682787 |
| CYC1 | 0.735362105 | 7.49105372 | 3.535011615 | 0.000518904 | 0.001584365 | -0.71378041 |
| UQCRH | 0.576764334 | 7.720106521 | 3.528655516 | 0.00053069 | 0.001584365 | -0.734545864 |
| PRDX3 | 0.634541712 | 6.651419371 | 3.528616662 | 0.000530762 | 0.001584365 | -0.734672699 |
| COX6A1 | 0.583941828 | 7.786369533 | 3.501245192 | 0.000584455 | 0.001718987 | -0.823723812 |
| NDUFA9 | 0.564150847 | 3.230789973 | 3.433428321 | 0.000740261 | 0.002117813 | -1.041756804 |
| MPC1 | -0.750046925 | 5.473253305 | -3.433048516 | 0.000741234 | 0.002117813 | -1.042967397 |
| IMMT | 0.504588674 | 6.341265293 | 3.427596691 | 0.000755336 | 0.002127706 | -1.060331692 |
| GOT2 | 0.663242603 | 6.894115645 | 3.394808701 | 0.00084558 | 0.002348833 | -1.164252937 |
| NDUFS8 | 0.660123022 | 5.776162946 | 3.312242477 | 0.00111945 | 0.003058295 | -1.422054704 |
| NDUFB6 | 0.566924319 | 5.749955746 | 3.309042874 | 0.001131569 | 0.003058295 | -1.431932414 |
| NDUFA7 | 0.52191043 | 3.10996661 | 3.281941505 | 0.001239235 | 0.003304626 | -1.515260483 |
| NDUFC2 | 0.566126775 | 5.071819697 | 3.244594948 | 0.001403285 | 0.003692855 | -1.629094903 |
| COX7A2L | 0.478197468 | 4.835521375 | 3.222036865 | 0.001511931 | 0.003927093 | -1.697293186 |
| TCIRG1 | 0.767848449 | 5.703195352 | 3.191075756 | 0.001673801 | 0.004291798 | -1.790206774 |
| VDAC2 | 0.646030667 | 7.078007642 | 3.143400612 | 0.001954747 | 0.004948727 | -1.931714346 |
| ATP6V1D | 0.513734804 | 4.939348093 | 3.119998659 | 0.002108082 | 0.005270206 | -2.000478517 |
| ETFA | 0.472896562 | 5.086285534 | 3.075758102 | 0.002428748 | 0.005961997 | -2.129216334 |
| NDUFB1 | 0.391488394 | 5.248462857 | 3.073737317 | 0.002444419 | 0.005961997 | -2.135057314 |
| COX17 | 0.618697316 | 4.952077829 | 3.035153867 | 0.002762093 | 0.006655647 | -2.245918529 |
| FH | 0.470348761 | 6.397952904 | 3.023516462 | 0.002865125 | 0.00681318 | -2.279108582 |
| BDH2 | -0.692315391 | 3.211368923 | -3.020147964 | 0.002895601 | 0.00681318 | -2.288694143 |
| COX6B1 | 0.513581519 | 8.614525587 | 3.011462145 | 0.002975568 | 0.006919926 | -2.313366467 |
| SDHB | 0.413221586 | 6.204804601 | 3.007708338 | 0.003010753 | 0.006921271 | -2.32400943 |
| FXN | 0.587789287 | 3.233874576 | 2.993294649 | 0.003149438 | 0.007157814 | -2.364764412 |
| GRPEL1 | 0.451304296 | 4.312181924 | 2.829569823 | 0.005192753 | 0.011669107 | -2.815222111 |
| ACO2 | -0.527129955 | 6.15080493 | -2.821730236 | 0.005315754 | 0.011812786 | -2.836212339 |
| CASP7 | 0.606466437 | 5.056109596 | 2.814038158 | 0.005439014 | 0.011906459 | -2.856755898 |
| ATP5MC2 | 0.433345876 | 6.635437808 | 2.811701372 | 0.005476971 | 0.011906459 | -2.862986703 |
| ATP5F1C | 0.437473021 | 7.681676649 | 2.793587879 | 0.005779471 | 0.01242897 | -2.911123907 |
| IDH1 | 0.772073137 | 6.166059099 | 2.778778396 | 0.006037995 | 0.012846798 | -2.950268991 |
| MAOB | -1.739160769 | 3.190359013 | -2.74381359 | 0.00669054 | 0.013961163 | -3.041932605 |
| NDUFV2 | 0.495374837 | 3.776262204 | 2.743260446 | 0.006701358 | 0.013961163 | -3.043374168 |
| UQCR10 | 0.36503588 | 7.226189148 | 2.737423122 | 0.006816495 | 0.014054628 | -3.058570693 |
| NDUFB5 | 0.499011293 | 5.246622846 | 2.686955998 | 0.007889156 | 0.016085227 | -3.188712158 |
| CS | 0.431093794 | 6.713150285 | 2.683750202 | 0.007962187 | 0.016085227 | -3.19690375 |
| IDH2 | -0.623563795 | 7.034662304 | -2.555092426 | 0.011446783 | 0.022893566 | -3.518189404 |
| COX15 | 0.414869418 | 5.026528647 | 2.539442246 | 0.011952705 | 0.023668722 | -3.556272957 |
| GLUD1 | 0.448361607 | 7.072157721 | 2.526667833 | 0.012380381 | 0.024275257 | -3.587197315 |
| NDUFA1 | 0.396218102 | 7.78670015 | 2.483065872 | 0.013944921 | 0.027077516 | -3.691655462 |
| COX11 | 0.409330058 | 4.021838123 | 2.477864073 | 0.014142863 | 0.027197813 | -3.704004373 |
| CYB5A | -0.663014805 | 3.327998007 | -2.464232463 | 0.01467349 | 0.027949505 | -3.736250776 |
| COX8A | 0.397408795 | 9.523453891 | 2.451869882 | 0.015169945 | 0.028622537 | -3.76535161 |
| NDUFA2 | 0.348455391 | 5.804272516 | 2.428686769 | 0.016141279 | 0.030170615 | -3.819554639 |
| ATP5MC1 | 0.446501755 | 5.748756541 | 2.384779895 | 0.018132962 | 0.03357956 | -3.920889655 |
| NDUFS3 | 0.338823584 | 5.140614123 | 2.380830724 | 0.018322321 | 0.033618937 | -3.929919241 |
| DLAT | 0.410683168 | 4.91526849 | 2.336409377 | 0.020575539 | 0.037410071 | -4.030518682 |
| SDHC | 0.35419457 | 3.84429928 | 2.327970367 | 0.021030127 | 0.037892121 | -4.049428977 |
| OXA1L | 0.343847585 | 6.090156274 | 2.272482198 | 0.024244852 | 0.043294379 | -4.172163262 |
| COX10 | 0.385920427 | 4.16644186 | 2.25191291 | 0.025541375 | 0.045205974 | -4.216951206 |
| DLST | 0.373788588 | 5.70891797 | 2.234060626 | 0.026715259 | 0.046868875 | -4.25551138 |
| NDUFA6 | 0.328244634 | 6.548333859 | 2.207313386 | 0.028561908 | 0.049672883 | -4.312741264 |
| NDUFB7 | 0.350530655 | 7.854606919 | 2.184186346 | 0.030247 | 0.05215 | -4.36169923 |
| ECI1 | 0.363166415 | 4.863157303 | 2.126992938 | 0.034788501 | 0.059467523 | -4.480672618 |
| MGST3 | 0.478155246 | 4.309758617 | 2.120969717 | 0.035299332 | 0.059829376 | -4.493027644 |
| RHOT1 | 0.341329801 | 4.194904234 | 2.114449254 | 0.035859597 | 0.06026823 | -4.506365095 |
| CYB5R3 | 0.345009034 | 6.992667036 | 2.062024905 | 0.040648368 | 0.067747281 | -4.612177585 |
| NDUFS2 | 0.311531042 | 6.023625379 | 2.001696679 | 0.046825632 | 0.077397738 | -4.730808074 |
| COX7B | 0.302571448 | 6.649606889 | 1.943701577 | 0.053497367 | 0.087700602 | -4.84167558 |
| SUCLA2 | 0.32593424 | 5.015277886 | 1.939056855 | 0.054064708 | 0.087910095 | -4.850419768 |
| SLC25A5 | 0.345431268 | 9.234021308 | 1.871286847 | 0.062934882 | 0.101507875 | -4.975720634 |
| NDUFB2 | 0.299031021 | 4.677080114 | 1.863397444 | 0.064042228 | 0.102467565 | -4.990029241 |
| DLD | 0.34911954 | 5.481825521 | 1.838972989 | 0.067573716 | 0.107118623 | -5.033957983 |
| NDUFS7 | 0.285938579 | 3.647420147 | 1.835960147 | 0.068020326 | 0.107118623 | -5.039338107 |
| ATP5F1B | 0.286311477 | 9.333056034 | 1.817244237 | 0.070849822 | 0.110702846 | -5.072569411 |
| ECHS1 | 0.331587343 | 7.215758893 | 1.805065498 | 0.072742775 | 0.112779496 | -5.094017384 |
| ETFB | 0.434382729 | 4.45495582 | 1.761120402 | 0.079923219 | 0.122958798 | -5.17025256 |
| NDUFA8 | 0.311744772 | 6.526557487 | 1.74932498 | 0.081946461 | 0.125109101 | -5.190406325 |
| ACADVL | -0.265612585 | 6.610604844 | -1.696092267 | 0.091604532 | 0.138794745 | -5.279731302 |
| SLC25A3 | 0.234355821 | 6.738894606 | 1.679376567 | 0.094820971 | 0.142587926 | -5.307229322 |
| NDUFV1 | 0.314970551 | 6.065087109 | 1.66568275 | 0.097523406 | 0.145274456 | -5.329559639 |
| ATP5MG | 0.270972613 | 6.447644611 | 1.661448906 | 0.098371396 | 0.145274456 | -5.336427854 |
| ATP5F1A | -0.342096776 | 6.299477098 | -1.659386433 | 0.09878663 | 0.145274456 | -5.339767498 |
| SUCLG1 | 0.260765729 | 5.355960966 | 1.627208557 | 0.105449471 | 0.152718951 | -5.391350243 |
| MTRF1 | 0.395581755 | 2.869585129 | 1.626012311 | 0.105703937 | 0.152718951 | -5.393248993 |
| ABCB7 | 0.257600216 | 4.488232214 | 1.623969266 | 0.106139671 | 0.152718951 | -5.396488697 |
| ATP6V1H | 0.234444415 | 4.753316001 | 1.597719737 | 0.111866789 | 0.158991916 | -5.437761335 |
| ACADM | -0.32343275 | 4.442223178 | -1.596721696 | 0.1120893 | 0.158991916 | -5.439317685 |
| NDUFS1 | -0.278447606 | 4.790228079 | -1.578475738 | 0.116219559 | 0.162229567 | -5.467603912 |
| COX5B | 0.282181836 | 7.903683889 | 1.577853131 | 0.116362596 | 0.162229567 | -5.468563548 |
| MFN2 | 0.312324797 | 6.343888582 | 1.575930023 | 0.116805288 | 0.162229567 | -5.471525348 |
| ALAS1 | 0.3262318 | 5.965006054 | 1.555143417 | 0.121675845 | 0.167828752 | -5.503314812 |
| MRPL34 | 0.251521553 | 5.619244721 | 1.529810875 | 0.127826482 | 0.17510477 | -5.541501124 |
| SLC25A20 | -0.464312362 | 3.712852503 | -1.505441034 | 0.133970938 | 0.182273385 | -5.577659784 |
| ISCA1 | 0.270620879 | 4.613884821 | 1.49758991 | 0.135998797 | 0.183579818 | -5.589188344 |
| UQCR11 | 0.213294264 | 6.095827847 | 1.494639651 | 0.136766964 | 0.183579818 | -5.593505301 |
| NDUFC1 | 0.234582774 | 5.419441243 | 1.469232886 | 0.143522603 | 0.191363471 | -5.630338025 |
| MRPL35 | 0.208496116 | 4.768478784 | 1.435812241 | 0.152798672 | 0.202382347 | -5.677849901 |
| AFG3L2 | 0.290892185 | 4.72584169 | 1.390787703 | 0.16601404 | 0.218439527 | -5.740168963 |
| UQCRB | 0.191755827 | 5.092637714 | 1.351959381 | 0.178091815 | 0.232799758 | -5.792351611 |
| ATP5ME | 0.257330344 | 7.059703413 | 1.342849917 | 0.181018611 | 0.234790428 | -5.804384561 |
| NDUFB8 | 0.182731006 | 5.010614223 | 1.339935408 | 0.181962582 | 0.234790428 | -5.80821759 |
| NNT | -0.365908267 | 4.614439179 | -1.30999529 | 0.191873852 | 0.245562302 | -5.847120745 |
| ATP1B1 | -0.502555373 | 8.275992996 | -1.307355961 | 0.192766407 | 0.245562302 | -5.850508855 |
| OAT | 0.297398114 | 6.288795322 | 1.295710571 | 0.196741341 | 0.249039672 | -5.865378018 |
| ATP5F1D | 0.226048183 | 6.704688853 | 1.231811763 | 0.219633007 | 0.276267934 | -5.944640701 |
| DECR1 | 0.202630807 | 5.082975802 | 1.207933419 | 0.228664257 | 0.284837371 | -5.973249371 |
| COX5A | 0.208549881 | 7.566283112 | 1.203239994 | 0.230470321 | 0.284837371 | -5.978807801 |
| PHYH | -0.393671429 | 3.946942244 | -1.201272497 | 0.231230462 | 0.284837371 | -5.981131578 |
| ATP5MC3 | 0.217037122 | 6.001780087 | 1.198918055 | 0.232142457 | 0.284837371 | -5.983907453 |
| NDUFA3 | 0.211950411 | 5.411153272 | 1.140522876 | 0.25559019 | 0.311695353 | -6.05103933 |
| AIFM1 | 0.19718336 | 5.204451047 | 1.110398096 | 0.26831493 | 0.325230218 | -6.084379929 |
| ECH1 | 0.234401247 | 6.688326727 | 1.068702028 | 0.286641131 | 0.34535076 | -6.129074562 |
| SDHA | 0.217929731 | 5.484072789 | 1.024174479 | 0.30713313 | 0.367824108 | -6.174939458 |
| HADHA | 0.144163804 | 7.109422379 | 1.01500062 | 0.311473687 | 0.370802009 | -6.184149284 |
| PDHX | 0.186282392 | 4.649523273 | 1.002945776 | 0.317239092 | 0.375430878 | -6.196126851 |
| SDHD | 0.165956924 | 6.460496023 | 0.988735398 | 0.324125469 | 0.381324081 | -6.210064427 |
| OGDH | 0.188799141 | 6.912357941 | 0.966443664 | 0.33512441 | 0.391958374 | -6.231531861 |
| NDUFA4 | 0.138012622 | 6.680732159 | 0.905890222 | 0.3662101 | 0.425825698 | -6.287399965 |
| CPT1A | 0.308083971 | 5.878852499 | 0.89311532 | 0.372992974 | 0.43120575 | -6.298729152 |
| UQCRFS1 | 0.192024936 | 5.913683688 | 0.886885592 | 0.376328944 | 0.432562005 | -6.304196035 |
| HADHB | -0.118369155 | 5.880674712 | -0.881683793 | 0.379128644 | 0.433289879 | -6.308731804 |
| ATP5PB | 0.127283756 | 6.751502599 | 0.875366065 | 0.382546284 | 0.434711686 | -6.314205052 |
| FDX1 | 0.166307678 | 4.465252202 | 0.853886733 | 0.394307499 | 0.445545196 | -6.332521382 |
| PDHB | -0.11789759 | 4.306390048 | -0.812099063 | 0.417812021 | 0.46945171 | -6.366862103 |
| COX7A2 | 0.112774192 | 7.296600536 | 0.729256475 | 0.466796661 | 0.521560515 | -6.429883532 |
| LDHB | 0.320014525 | 7.188855525 | 0.724230816 | 0.469868292 | 0.52207588 | -6.433490161 |
| NDUFS4 | 0.114584583 | 5.74873619 | 0.638105596 | 0.524219349 | 0.579247899 | -6.491441679 |
| ACAA1 | -0.116494801 | 3.608039231 | -0.573336486 | 0.567135517 | 0.623225843 | -6.530217493 |
| COX4I1 | 0.087502628 | 7.138321388 | 0.563277553 | 0.573949868 | 0.625910682 | -6.535869017 |
| POLR2F | -0.258069443 | #NAME? | -0.560378798 | 0.575945056 | 0.625910682 | -6.536256956 |
| SLC25A6 | -0.124791514 | 8.848112927 | -0.555907462 | 0.57896738 | 0.625910682 | -6.53994657 |
| MDH1 | 0.071133553 | 5.799696305 | 0.527171786 | 0.598726179 | 0.64379159 | -6.5553336 |
| UQCRQ | -0.079019889 | 7.017964544 | -0.502754593 | 0.615753997 | 0.658560424 | -6.567768554 |
| ISCU | -0.069651152 | 5.245179041 | -0.490426847 | 0.624431707 | 0.66428905 | -6.573823363 |
| RETSAT | -0.109865068 | 5.493685903 | -0.469920978 | 0.638982702 | 0.676172171 | -6.583562806 |
| SLC25A12 | 0.120195519 | 3.837364705 | 0.462663226 | 0.644166966 | 0.676200847 | -6.586910533 |
| NDUFA5 | -0.074577602 | 3.968586242 | -0.460421483 | 0.645771809 | 0.676200847 | -6.587934058 |
| ATP5PF | -0.046567686 | 6.599266217 | -0.299774715 | 0.76469636 | 0.796558708 | -6.648359665 |
| SURF1 | -0.045918343 | 5.309144676 | -0.286567138 | 0.774774753 | 0.802875392 | -6.652192826 |
| UQCRC2 | 0.040041457 | 6.323566673 | 0.274346287 | 0.784134451 | 0.808386032 | -6.655585937 |
| ACAA2 | -0.105886025 | 4.062835072 | -0.231846726 | 0.816921547 | 0.837868253 | -6.666235612 |
| ATP5PO | -0.024899298 | 5.017534726 | -0.171182037 | 0.864273728 | 0.881911967 | -6.678340597 |
| COX7C | -0.01995104 | 7.537914438 | -0.137047336 | 0.891147199 | 0.904717969 | -6.683550209 |
| UQCRC1 | -0.019522863 | 7.191036995 | -0.124744447 | 0.900865663 | 0.909965316 | -6.685145042 |
| PDHA1 | -0.01695557 | 5.524613426 | -0.095361988 | 0.924133838 | 0.928777727 | -6.688347511 |
| BCKDHA | 0.011451183 | 1.609593431 | 0.050619664 | 0.95968496 | 0.95968496 | -6.691581934 |
