# Supplementary material for: A prognosis model for predicting immunotherapy response of esophageal cancer based on oxidative stress-related signatures
Source: Oncol Res. 2023 Nov 15;32(1):199–212. doi: 10.32604/or.2023.030969 (PMC10774069; doi:10.32604/or.2023.030969)
Supplement: Table S3 [file OncolRes-32-30969-s003.docx]

Table S3 Results of differential analysis of oxidative stress genes in the GEO dataset

| Tag | logFC | AveExpr | *t* | *p* Value | adj.*p*.Val | B |
| --- | --- | --- | --- | --- | --- | --- |
| HADHB | -0.962811014 | 14.28132041 | -30.94682235 | 5.96E-104 | 1.13E-101 | 226.7716951 |
| PDK4 | -3.163559399 | 12.04225322 | -29.18997098 | 2.33E-97 | 2.20E-95 | 211.6388132 |
| ACADM | -1.385664648 | 13.65340404 | -26.53692391 | 4.21E-87 | 2.65E-85 | 188.08189 |
| ACADVL | -1.094894897 | 15.20575713 | -20.31830747 | 6.96E-62 | 3.29E-60 | 130.1520858 |
| SLC25A4 | -1.290708101 | 9.564373438 | -18.11726914 | 9.54E-53 | 3.60E-51 | 109.1676439 |
| MPC1 | -1.276171618 | 10.4886814 | -17.73011899 | 3.84E-51 | 1.21E-49 | 105.4810926 |
| ACAA1 | -1.538457038 | 13.48993634 | -17.09695582 | 1.60E-48 | 4.33E-47 | 99.46510537 |
| ETFDH | -0.885937061 | 11.08801642 | -15.07988989 | 2.93E-40 | 6.91E-39 | 80.50584779 |
| SUCLG1 | -0.727170824 | 13.50736551 | -14.88615605 | 1.78E-39 | 3.73E-38 | 78.70946725 |
| LDHB | 0.982734359 | 14.18068783 | 14.23698658 | 7.17E-37 | 1.35E-35 | 72.73318564 |
| ATP6V1C1 | 0.941959899 | 12.20741146 | 14.07740892 | 3.10E-36 | 5.32E-35 | 71.27532347 |
| ACADSB | -1.107486396 | 9.760574174 | -13.85178839 | 2.43E-35 | 3.83E-34 | 69.22242185 |
| MAOB | -1.816000903 | 10.42182525 | -13.49052043 | 6.47E-34 | 9.41E-33 | 65.95694223 |
| ATP5PB | -0.741043339 | 11.35281792 | -13.45964309 | 8.55E-34 | 1.15E-32 | 65.67914815 |
| ATP5PO | -0.630398533 | 14.63101456 | -12.95352007 | 8.04E-32 | 1.01E-30 | 61.15724178 |
| HADHA | -0.623103374 | 13.9434568 | -12.83159251 | 2.38E-31 | 2.81E-30 | 60.07725321 |
| COX7C | -0.636069249 | 15.01978563 | -12.64779821 | 1.21E-30 | 1.35E-29 | 58.45658211 |
| NDUFC1 | -0.551331464 | 13.68836434 | -12.61011091 | 1.69E-30 | 1.78E-29 | 58.12537523 |
| ATP5F1A | -0.71191037 | 14.57940467 | -11.87139358 | 1.07E-27 | 1.06E-26 | 51.71544125 |
| OPA1 | 0.421413723 | 11.54380621 | 11.81138446 | 1.79E-27 | 1.69E-26 | 51.20201348 |
| BCKDHA | -0.996676723 | 12.04095567 | -11.59116772 | 1.18E-26 | 1.06E-25 | 49.32791254 |
| ACAT1 | -0.97539721 | 12.04524406 | -11.52929278 | 2.00E-26 | 1.72E-25 | 48.80424418 |
| FDX1 | -0.99597527 | 10.02857036 | -11.44172775 | 4.20E-26 | 3.45E-25 | 48.06538122 |
| MTRR | 0.523462163 | 11.30711676 | 11.41457512 | 5.29E-26 | 4.17E-25 | 47.83680737 |
| OAT | -0.635726466 | 13.64056419 | -11.40440133 | 5.77E-26 | 4.36E-25 | 47.75122927 |
| NDUFS1 | -0.510512786 | 12.19344967 | -11.37236653 | 7.56E-26 | 5.50E-25 | 47.48200035 |
| MRPS30 | 0.560347908 | 13.32151644 | 11.24817803 | 2.16E-25 | 1.51E-24 | 46.44170775 |
| ATP5PF | -0.54038695 | 14.39768941 | -11.24286 | 2.25E-25 | 1.52E-24 | 46.39728276 |
| UQCRB | -0.835934468 | 9.495424503 | -11.10093835 | 7.41E-25 | 4.83E-24 | 45.21550297 |
| VDAC2 | -0.607845578 | 14.02988532 | -10.94885554 | 2.63E-24 | 1.66E-23 | 43.95736156 |
| ATP5ME | -0.536935282 | 14.86314529 | -10.77148609 | 1.14E-23 | 6.95E-23 | 42.50112593 |
| ECHS1 | -0.662729874 | 13.41139105 | -10.63198223 | 3.58E-23 | 2.12E-22 | 41.36440559 |
| UQCRQ | -0.612217218 | 14.31774115 | -10.35525359 | 3.40E-22 | 1.95E-21 | 39.13282259 |
| MRPL11 | 0.702259897 | 12.71065941 | 10.27195936 | 6.64E-22 | 3.69E-21 | 38.46736374 |
| PHB2 | 0.573193803 | 13.64101774 | 10.03114944 | 4.55E-21 | 2.45E-20 | 36.56026621 |
| ATP5MG | -0.549042369 | 13.48045365 | -10.00663283 | 5.52E-21 | 2.90E-20 | 36.3675335 |
| CASP7 | 0.683086679 | 11.06651777 | 9.956197309 | 8.23E-21 | 4.20E-20 | 35.97188773 |
| ATP6V0C | -0.504770568 | 13.97035106 | -9.810902046 | 2.58E-20 | 1.28E-19 | 34.8385288 |
| TIMM50 | 0.479832113 | 11.44608473 | 9.764450624 | 3.71E-20 | 1.80E-19 | 34.47822748 |
| ALDH6A1 | -0.653746803 | 10.97759362 | -9.676332154 | 7.38E-20 | 3.49E-19 | 33.79748819 |
| SDHD | -0.617248731 | 12.79633669 | -9.498479636 | 2.92E-19 | 1.35E-18 | 32.43469312 |
| HTRA2 | 0.578595374 | 11.73238771 | 9.220821963 | 2.43E-18 | 1.09E-17 | 30.33783896 |
| MRPL15 | 0.623261257 | 12.79967191 | 9.207429599 | 2.69E-18 | 1.18E-17 | 30.23766937 |
| IDH2 | -0.574004603 | 13.63289575 | -8.998092002 | 1.29E-17 | 5.55E-17 | 28.68378592 |
| SLC25A20 | -0.664472723 | 10.73293341 | -8.967079821 | 1.63E-17 | 6.84E-17 | 28.4555096 |
| BAX | 0.518211591 | 11.14014888 | 8.93213809 | 2.11E-17 | 8.67E-17 | 28.19891084 |
| TIMM17A | 0.512901277 | 12.83722372 | 8.910756083 | 2.47E-17 | 9.94E-17 | 28.04220566 |
| ACAA2 | -0.750460598 | 12.87801476 | -8.495162731 | 5.13E-16 | 2.02E-15 | 25.04514521 |
| COX7A2L | 0.36454619 | 11.21132247 | 8.429212955 | 8.22E-16 | 3.17E-15 | 24.5782749 |
| IMMT | 0.35399986 | 10.83679192 | 8.267444882 | 2.59E-15 | 9.81E-15 | 23.44351455 |
| MRPS15 | 0.462485992 | 13.91802921 | 8.174717449 | 4.98E-15 | 1.85E-14 | 22.7998307 |
| GPX4 | 0.529897969 | 14.50750824 | 8.113976951 | 7.62E-15 | 2.77E-14 | 22.38090331 |
| FXN | 0.532060091 | 11.35822045 | 7.843307757 | 4.92E-14 | 1.75E-13 | 20.54069426 |
| ATP6V0B | 0.491122131 | 12.97976299 | 7.80027523 | 6.59E-14 | 2.31E-13 | 20.25219451 |
| COX6C | 0.420952708 | 11.75505484 | 7.564879509 | 3.21E-13 | 1.10E-12 | 18.69425431 |
| ATP6V1D | -0.665318564 | 7.744627241 | -7.519848963 | 4.32E-13 | 1.46E-12 | 18.40016982 |
| SLC25A11 | 0.400003673 | 12.28142678 | 7.505602718 | 4.75E-13 | 1.57E-12 | 18.30739776 |
| COX6B1 | -0.495127271 | 14.3954771 | -7.50116892 | 4.89E-13 | 1.59E-12 | 18.27855096 |
| NDUFS6 | 0.406515469 | 14.35633758 | 7.33156242 | 1.49E-12 | 4.76E-12 | 17.18449929 |
| ISCA1 | 0.425401638 | 11.30643929 | 7.323661861 | 1.56E-12 | 4.93E-12 | 17.13398766 |
| ATP5F1D | -0.682799126 | 14.84072212 | -7.260568605 | 2.35E-12 | 7.30E-12 | 16.73206028 |
| COX17 | 0.723196891 | 13.53545602 | 7.217390161 | 3.11E-12 | 9.33E-12 | 16.45849446 |
| GPI | 0.409803757 | 14.26634081 | 7.217361406 | 3.11E-12 | 9.33E-12 | 16.45831268 |
| DECR1 | -0.548767112 | 12.79983281 | -7.119464926 | 5.82E-12 | 1.72E-11 | 15.84261234 |
| PDP1 | 0.571846134 | 12.4655774 | 7.041209171 | 9.56E-12 | 2.78E-11 | 15.35500911 |
| ATP6V1F | -0.417744103 | 14.26956981 | -7.012361017 | 1.15E-11 | 3.28E-11 | 15.17629184 |
| NDUFB3 | -0.32334464 | 13.57484374 | -6.962543099 | 1.57E-11 | 4.42E-11 | 14.86898206 |
| ALAS1 | -0.382710539 | 12.27171495 | -6.947217562 | 1.73E-11 | 4.80E-11 | 14.77478082 |
| LRPPRC | 0.298436332 | 11.91613107 | 6.911652812 | 2.16E-11 | 5.90E-11 | 14.55678834 |
| DLAT | -0.324214761 | 11.30817194 | -6.853057928 | 3.10E-11 | 8.37E-11 | 14.19950947 |
| TCIRG1 | 0.27040264 | 11.84543549 | 6.787987495 | 4.63E-11 | 1.23E-10 | 13.80549819 |
| PDHB | -0.618173992 | 12.94833406 | -6.763362276 | 5.39E-11 | 1.42E-10 | 13.65714824 |
| UQCRC2 | -0.350347807 | 14.5361894 | -6.74435962 | 6.06E-11 | 1.57E-10 | 13.54295653 |
| RETSAT | -0.292025433 | 9.861343897 | -6.701241552 | 7.88E-11 | 2.01E-10 | 13.28477682 |
| LDHA | 0.401885082 | 12.46366613 | 6.664134325 | 9.88E-11 | 2.49E-10 | 13.06362301 |
| VDAC1 | -0.228334586 | 14.2169585 | -6.606689057 | 1.40E-10 | 3.48E-10 | 12.7231543 |
| SLC25A6 | -0.33323074 | 14.83178538 | -6.461163343 | 3.34E-10 | 8.19E-10 | 11.87105113 |
| TIMM8B | -0.558925974 | 13.02866686 | -6.347583585 | 6.51E-10 | 1.58E-09 | 11.21648378 |
| COX4I1 | -0.256283735 | 14.65004493 | -6.302921841 | 8.45E-10 | 2.02E-09 | 10.96163886 |
| HSD17B10 | -0.473463885 | 14.09389904 | -6.271631227 | 1.01E-09 | 2.39E-09 | 10.78395199 |
| SLC25A5 | 0.314527992 | 14.95250163 | 6.178593917 | 1.73E-09 | 4.04E-09 | 10.25984426 |
| ETFB | -0.49327336 | 14.43156254 | -6.16073062 | 1.92E-09 | 4.42E-09 | 10.15993996 |
| MRPS12 | 0.590293897 | 11.75941433 | 5.951297412 | 6.26E-09 | 1.43E-08 | 9.006250023 |
| CYB5A | -0.516804399 | 13.80847336 | -5.932836741 | 6.94E-09 | 1.56E-08 | 8.906125848 |
| COX7B | -0.313346573 | 13.94643433 | -5.875853856 | 9.51E-09 | 2.11E-08 | 8.598688597 |
| ATP5F1C | -0.262895279 | 12.88803254 | -5.866276182 | 1.00E-08 | 2.20E-08 | 8.54725515 |
| NDUFB8 | -0.323166887 | 13.65347754 | -5.743276731 | 1.96E-08 | 4.26E-08 | 7.892921155 |
| CS | -0.329109606 | 13.76619653 | -5.731889759 | 2.09E-08 | 4.48E-08 | 7.832928117 |
| SDHC | -0.211431885 | 11.35528099 | -5.588901427 | 4.49E-08 | 9.53E-08 | 7.088073863 |
| COX7A2 | -0.354525869 | 14.20551767 | -5.565878934 | 5.07E-08 | 1.06E-07 | 6.969623072 |
| UQCR11 | -0.321062168 | 14.99017804 | -5.563214425 | 5.14E-08 | 1.07E-07 | 6.955940764 |
| ATP6AP1 | -0.305167751 | 13.48859541 | -5.382587472 | 1.32E-07 | 2.71E-07 | 6.04136463 |
| ATP5PD | -0.295593584 | 14.03593832 | -5.357432086 | 1.50E-07 | 3.05E-07 | 5.916031077 |
| GOT2 | 0.283307726 | 14.26495273 | 5.329850261 | 1.73E-07 | 3.47E-07 | 5.779184323 |
| NDUFA8 | 0.409697492 | 13.62661802 | 5.264586808 | 2.41E-07 | 4.79E-07 | 5.457789404 |
| PMPCA | 0.242421074 | 8.171694329 | 5.232057207 | 2.84E-07 | 5.59E-07 | 5.298863908 |
| CYCS | 0.322937701 | 13.31425004 | 5.184213243 | 3.61E-07 | 7.03E-07 | 5.066659446 |
| AIFM1 | 0.294429422 | 11.44870358 | 5.091698675 | 5.71E-07 | 1.10E-06 | 4.622878801 |
| HSPA9 | 0.230609307 | 13.67782442 | 5.061858992 | 6.61E-07 | 1.26E-06 | 4.481218194 |
| RHOT1 | -0.191973715 | 11.02993733 | -5.051481579 | 6.95E-07 | 1.31E-06 | 4.432121973 |
| ATP6V1E1 | -0.397179779 | 12.67913838 | -4.994651685 | 9.17E-07 | 1.72E-06 | 4.16481044 |
| SDHA | 0.2065342 | 12.47123804 | 4.985377436 | 9.59E-07 | 1.78E-06 | 4.121437056 |
| MRPS22 | 0.205011236 | 10.88348409 | 4.900429164 | 1.44E-06 | 2.65E-06 | 3.727432441 |
| ATP5MF | 0.318549855 | 14.28509736 | 4.885586573 | 1.55E-06 | 2.81E-06 | 3.659198256 |
| NNT | 0.304012453 | 10.51777236 | 4.872605349 | 1.65E-06 | 2.96E-06 | 3.599669902 |
| GLUD1 | -0.261902743 | 13.59639032 | -4.860367649 | 1.75E-06 | 3.11E-06 | 3.543678406 |
| COX8A | -0.295475444 | 14.77140222 | -4.850758617 | 1.83E-06 | 3.23E-06 | 3.499800573 |
| NDUFS4 | -0.435347075 | 13.1125979 | -4.833417585 | 1.98E-06 | 3.47E-06 | 3.420809106 |
| NDUFS8 | 0.368392434 | 14.44047463 | 4.818121552 | 2.13E-06 | 3.70E-06 | 3.35133945 |
| CPT1A | 0.428701824 | 12.29342178 | 4.787264806 | 2.46E-06 | 4.23E-06 | 3.211788061 |
| NDUFA4 | 0.240414536 | 15.24908441 | 4.581664434 | 6.35E-06 | 1.08E-05 | 2.302221125 |
| ATP6V1H | -0.345525268 | 11.64128538 | -4.340674013 | 1.84E-05 | 3.11E-05 | 1.281510087 |
| NDUFAB1 | 0.294841701 | 13.13830457 | 4.300934557 | 2.19E-05 | 3.66E-05 | 1.117958516 |
| UQCRFS1 | -0.301699109 | 13.13800452 | -4.283676771 | 2.36E-05 | 3.91E-05 | 1.04735548 |
| SUPV3L1 | 0.421557802 | 12.10192459 | 4.276601295 | 2.43E-05 | 3.99E-05 | 1.018483279 |
| NDUFB5 | 0.207894147 | 13.47489043 | 4.180661963 | 3.64E-05 | 5.94E-05 | 0.631261832 |
| ETFA | -0.28476433 | 13.61564739 | -4.159442176 | 3.98E-05 | 6.44E-05 | 0.546693365 |
| NDUFB1 | -0.207878154 | 13.0485085 | -4.1244832 | 4.61E-05 | 7.32E-05 | 0.40822294 |
| SLC25A3 | -0.184825254 | 14.33682298 | -4.12442406 | 4.61E-05 | 7.32E-05 | 0.40798959 |
| NDUFA5 | -0.251785358 | 13.74442473 | -4.100593215 | 5.09E-05 | 8.01E-05 | 0.314208315 |
| ISCU | -0.228563877 | 13.95254932 | -4.097529411 | 5.15E-05 | 8.05E-05 | 0.302187291 |
| NDUFA2 | -0.295368318 | 13.15814916 | -4.004414113 | 7.54E-05 | 0.000116787 | -0.059240869 |
| COX5B | -0.242523941 | 14.13690665 | -3.875122228 | 0.000126389 | 0.000194208 | -0.548454728 |
| DLST | -0.19004333 | 12.53240475 | -3.783016295 | 0.000181092 | 0.000276019 | -0.887944477 |
| TIMM13 | -0.268550412 | 7.888337134 | -3.743246691 | 0.00021105 | 0.000319108 | -1.032197139 |
| NDUFV1 | 0.216952966 | 14.4592009 | 3.611899225 | 0.000346643 | 0.000519965 | -1.498571605 |
| IDH1 | -0.255056017 | 12.93168618 | -3.579593134 | 0.00039077 | 0.000581539 | -1.610906824 |
| MGST3 | -0.227947605 | 13.76193787 | -3.505685063 | 0.000512311 | 0.00075646 | -1.864360012 |
| CYC1 | 0.233905818 | 14.57520195 | 3.493015814 | 0.000536406 | 0.000785898 | -1.907310789 |
| UQCRC1 | -0.226711941 | 14.55442028 | -3.489697165 | 0.000542891 | 0.00078928 | -1.918537507 |
| TIMM9 | 0.282970057 | 10.59148007 | 3.465662626 | 0.000592089 | 0.000854236 | -1.999546429 |
| TIMM10 | 0.374093212 | 13.20957022 | 3.336777286 | 0.00093496 | 0.001334415 | -2.424997846 |
| MTX2 | 0.199078095 | 13.38110646 | 3.335531692 | 0.000939033 | 0.001334415 | -2.429035666 |
| RHOT2 | 0.337238561 | 11.18953322 | 3.320809911 | 0.000988433 | 0.001394133 | -2.476651563 |
| NDUFA1 | -0.156458352 | 14.4389428 | -3.291726077 | 0.001093203 | 0.001530485 | -2.570137027 |
| NDUFA3 | -0.187834196 | 13.57208245 | -3.2219107 | 0.001388214 | 0.001929209 | -2.791382591 |
| TOMM22 | 0.187810003 | 13.63099988 | 2.963054264 | 0.003246353 | 0.004478545 | -3.572456169 |
| ATP6V1G1 | -0.158504835 | 14.5308051 | -2.923345301 | 0.003679514 | 0.005039334 | -3.68677107 |
| POR | 0.110095104 | 13.44133609 | 2.799660464 | 0.005388377 | 0.007326642 | -4.03339232 |
| NDUFB2 | -0.157601479 | 13.97243732 | -2.689115496 | 0.00749382 | 0.010116658 | -4.331036811 |
| PHYH | -0.227961989 | 11.67444765 | -2.641393678 | 0.008612598 | 0.011544546 | -4.455968712 |
| CYB5R3 | 0.093040696 | 14.49443282 | 2.534388921 | 0.011682875 | 0.015549743 | -4.728264401 |
| NQO2 | 0.093928184 | 9.128080063 | 2.496078946 | 0.01299929 | 0.017114525 | -4.823110627 |
| ATP5F1E | -0.12929568 | 10.66145382 | -2.494960187 | 0.013039638 | 0.017114525 | -4.825859431 |
| NDUFS3 | -0.132796668 | 12.31640391 | -2.274885871 | 0.023493971 | 0.030623176 | -5.343359792 |
| DLD | 0.126097155 | 8.667843624 | 2.243991391 | 0.025433608 | 0.032924328 | -5.412296144 |
| VDAC3 | -0.13308505 | 13.68218689 | -2.214321062 | 0.027425874 | 0.035261838 | -5.477637775 |
| COX6A1 | -0.090286626 | 14.53047276 | -2.166717288 | 0.03090482 | 0.03946629 | -5.58070403 |
| OGDH | -0.140085749 | 12.03877622 | -2.162072906 | 0.031263771 | 0.03965673 | -5.59064268 |
| UQCRH | -0.138142944 | 13.00614359 | -2.13100114 | 0.033758863 | 0.042536167 | -5.656599207 |
| MDH1 | -0.100027817 | 13.85074154 | -1.995288328 | 0.046756636 | 0.058523207 | -5.933748259 |
| NDUFB4 | 0.087992218 | 14.53939416 | 1.913617885 | 0.05645327 | 0.070195184 | -6.091935892 |
| HCCS | -0.10837208 | 11.2941562 | -1.853949652 | 0.064555331 | 0.079744821 | -6.203411121 |
| PRDX3 | 0.140856545 | 12.78218389 | 1.832682279 | 0.06766672 | 0.08304552 | -6.24230643 |
| ATP5F1B | -0.116790137 | 14.88618009 | -1.80919619 | 0.071245584 | 0.086873647 | -6.284747546 |
| ATP5MC1 | 0.104892053 | 13.05812438 | 1.692655327 | 0.091376971 | 0.110706715 | -6.487384678 |
| OXA1L | 0.084835592 | 13.65559778 | 1.65096392 | 0.099608619 | 0.119911013 | -6.556652403 |
| MFN2 | 0.080516426 | 12.66697777 | 1.580587093 | 0.114841217 | 0.137373355 | -6.669716785 |
| IDH3B | 0.057715686 | 12.0590237 | 1.528847147 | 0.127171065 | 0.151165605 | -6.749741268 |
| ABCB7 | 0.082745425 | 12.06444971 | 1.469410161 | 0.142585522 | 0.168429148 | -6.838424706 |
| MRPL34 | 0.074301556 | 13.76909927 | 1.422721056 | 0.155673663 | 0.182747343 | -6.905650575 |
| NDUFB6 | -0.106625788 | 13.35235359 | -1.292361868 | 0.197051866 | 0.229893843 | -7.081977338 |
| ATP5MC3 | 0.058718811 | 12.40500333 | 1.261284746 | 0.208014206 | 0.241194386 | -7.121537014 |
| FH | -0.106327282 | 12.09966652 | -1.24208276 | 0.21500589 | 0.247781178 | -7.145503131 |
| IDH3G | 0.058682721 | 13.59759636 | 1.208047613 | 0.227813414 | 0.26094991 | -7.187086718 |
| AFG3L2 | -0.087115425 | 11.42638365 | -1.196103267 | 0.232435004 | 0.264639854 | -7.201408463 |
| NDUFA7 | 0.126608568 | 11.75802001 | 1.175219197 | 0.24067538 | 0.272381118 | -7.226110012 |
| SUCLA2 | -0.053364286 | 8.623197697 | -1.146279229 | 0.252432799 | 0.283986899 | -7.259626177 |
| MDH2 | 0.048005302 | 14.49354193 | 0.940239526 | 0.347718391 | 0.388868497 | -7.474239732 |
| GRPEL1 | 0.062934098 | 11.83890171 | 0.918103617 | 0.359172708 | 0.39931554 | -7.494788596 |
| ATP5MC2 | 0.037942943 | 15.02592542 | 0.900706512 | 0.368340062 | 0.4071127 | -7.510596428 |
| PDHA1 | 0.032470118 | 10.13318656 | 0.827533283 | 0.40847719 | 0.448849935 | -7.573787896 |
| COX10 | 0.068687764 | 9.364584666 | 0.782915562 | 0.434185882 | 0.471682053 | -7.609702807 |
| TOMM70 | -0.058596765 | 11.3052868 | -0.782811414 | 0.43424697 | 0.471682053 | -7.609784321 |
| NDUFB7 | -0.069090363 | 14.26505977 | -0.761312682 | 0.446963383 | 0.482720453 | -7.626379364 |
| SDHB | 0.047580659 | 13.05353993 | 0.734353681 | 0.46320611 | 0.497420197 | -7.646538212 |
| SURF1 | -0.054297606 | 11.89087331 | -0.654124649 | 0.513444653 | 0.54825446 | -7.702242467 |
| PDHX | -0.03764357 | 11.71054252 | -0.644320342 | 0.519773635 | 0.551894478 | -7.708609429 |
| NDUFA6 | 0.032323788 | 14.50196456 | 0.553424022 | 0.580312935 | 0.610173149 | -7.763069073 |
| SLC25A12 | -0.035738303 | 12.23322416 | -0.552248249 | 0.581117285 | 0.610173149 | -7.763719472 |
| MTRF1 | -0.031609306 | 10.49761498 | -0.469229855 | 0.639186346 | 0.667437676 | -7.806150633 |
| POLR2F | 0.016852537 | 10.44265038 | 0.43864861 | 0.661176467 | 0.686606331 | -7.820044772 |
| UQCR10 | -0.021140489 | 12.50757576 | -0.387394645 | 0.698690536 | 0.721598423 | -7.841234279 |
| COX15 | 0.021197704 | 10.41166669 | 0.377843097 | 0.705767441 | 0.724945904 | -7.844892672 |
| COX11 | -0.022424788 | 9.972182089 | -0.366479123 | 0.714220566 | 0.729663173 | -7.84912638 |
| ECI1 | 0.02214095 | 13.60078096 | 0.251818926 | 0.801323178 | 0.814247746 | -7.884614879 |
| NDUFS2 | -0.01061481 | 13.12242345 | -0.142650253 | 0.886645354 | 0.896128192 | -7.906175251 |
| ATP6V0E1 | -0.004181933 | 14.32966783 | -0.11797618 | 0.906151621 | 0.910971576 | -7.909395089 |
| NDUFC2 | -0.003060674 | 12.06967433 | -0.039837916 | 0.968244194 | 0.968244194 | -7.915569499 |
